# Integration of Face-to-Face Screening With Real-time Machine Learning to Predict Risk of Suicide Among Adults

**DOI:** 10.1001/jamanetworkopen.2022.12095

**Published:** 2022-05-13

**Authors:** Drew Wilimitis, Robert W. Turer, Michael Ripperger, Allison B. McCoy, Sarah H. Sperry, Elliot M. Fielstein, Troy Kurz, Colin G. Walsh

**Affiliations:** 1 Department of Biomedical Informatics, Vanderbilt University Medical Center, Nashville, Tennessee; 2 Department of Psychiatry and Behavioral Sciences, Vanderbilt University Medical Center, Nashville, Tennessee; 3 Department of Medicine, Vanderbilt University Medical Center, Nashville, Tennessee

## Abstract

**Importance:**

Understanding the differences and potential synergies between traditional clinician assessment and automated machine learning might enable more accurate and useful suicide risk detection.

**Objective:**

To evaluate the respective and combined abilities of a real-time machine learning model and the Columbia Suicide Severity Rating Scale (C-SSRS) to predict suicide attempt (SA) and suicidal ideation (SI).

**Design, Setting, and Participants:**

This cohort study included encounters with adult patients (aged ≥18 years) at a major academic medical center. The C-SSRS was administered during routine care, and a Vanderbilt Suicide Attempt and Ideation Likelihood (VSAIL) prediction was generated in the electronic health record. Encounters took place in the inpatient, ambulatory surgical, and emergency department settings. Data were collected from June 2019 to September 2020.

**Main Outcomes and Measures:**

Primary outcomes were the incidence of SA and SI, encoded as *International Classification of Diseases* codes, occurring within various time periods after an index visit. We evaluated the retrospective validity of the C-SSRS, VSAIL, and ensemble models combining both. Discrimination metrics included area under the receiver operating curve (AUROC), area under the precision-recall curve (AUPR), sensitivity, specificity, positive predictive value (PPV), and negative predictive value (NPV).

**Results:**

The cohort included 120 398 unique index visits for 83 394 patients (mean [SD] age, 51.2 [20.6] years; 38 107 [46%] men; 45 273 [54%] women; 13 644 [16%] Black; 63 869 [77%] White). Within 30 days of an index visit, the combined models had higher AUROC (SA: 0.874-0.887; SI: 0.869-0.879) than both the VSAIL (SA: 0.729; SI: 0.773) and C-SSRS (SA: 0.823; SI: 0.777) models. In the highest risk-decile, ensemble methods had PPV of 1.3% to 1.4% for SA and 8.3% to 8.7% for SI and sensitivity of 77.6% to 79.5% for SA and 67.4% to 70.1% for SI, outperforming VSAIL (PPV for SA: 0.4%; PPV for SI: 3.9%; sensitivity for SA: 28.8%; sensitivity for SI: 35.1%) and C-SSRS (PPV for SA: 0.5%; PPV for SI: 3.5%; sensitivity for SA: 76.6%; sensitivity for SI: 68.8%).

**Conclusions and Relevance:**

In this study, suicide risk prediction was optimal when leveraging both in-person screening (for acute measures of risk in patient-reported suicidality) and historical EHR data (for underlying clinical factors that can quantify a patient’s passive risk level). To improve suicide risk classification, prediction systems could combine pretrained machine learning with structured clinician assessment without needing to retrain the original model.

## Key Points

**Question:**

Does prediction of suicide risk improve when combining face-to-face screening with electronic health record–based machine learning models?

**Findings:**

In this cohort study of 120 398 adult patient encounters, an ensemble learning approach combined suicide risk predictions from the Columbia Suicide Severity Rating Scale and a real-time machine learning model. Combined models outperformed either model alone for risks of suicide attempt and suicidal ideation across a variety of time periods.

**Meaning:**

These findings suggest that health care systems should attempt to leverage the independent, complementary strengths of traditional clinician assessment and automated machine learning to improve suicide risk detection.

## Teaser

This cohort study evaluates the respective and combined abilities of a real-time machine learning model and the Columbia Suicide Severity Rating Scale to predict suicide attempt and suicidal ideation among adults.

## Introduction

Nearly 800 000 people die from suicide annually worldwide, with anticipated worsening during the COVID-19 pandemic.^1,2^ Rates of suicidal behavior are increasing in the United States, although preventive measures including universal screening with discharge follow-up, lethal means counseling, and safety planning have been shown to reduce risk.^3,4,5,6^ In the month prior to their death, approximately 45% of individuals had seen a primary care clinician and 19% had seen a mental health specialist.^7^ Mental health diagnoses are often absent in records of those who subsequently die from suicide, and many patients will not proactively disclose suicidal thoughts and behaviors because of stigma.^8,9^ Better risk identification and prognostication might improve outreach and prevention.

Universal screening in emergency departments (EDs) has demonstrated feasibility and led to a near 2-fold increase in suicide risk detection in adults.^4^ Despite the challenges of implementing universal suicide risk screening (eg, additional training requirements, competing medical priorities, and limited availability of mental health resources), it is often recommended for health care settings, particularly primary care, medical specialty clinics, and EDs.^10,11^ Endorsed by the US Centers for Disease Control and Prevention in 2011 and the US Food and Drug Administration in 2012, the Columbia Suicide Severity Rating Scale (C-SSRS) is a standardized assessment of suicidal ideation and behavior.^12^ In a multisite analysis of existing suicide risk scales, Posner et al^13^ found the C-SSRS to have comparatively high sensitivity and predictive validity in the classification of both historical and future suicidal ideation and behavior.^13^ Others have criticized the score as overly simplified with the potential to miss a subset of patients with suicidal ideation.^14^

Predictive models might augment face-to-face screening and leverage electronic health record (EHR) data toward the automated, early detection of individuals at risk of suicide. Kessler et al^15^ used administrative data to predict deaths from suicide following outpatient mental health visits in an active-duty military population. In a civilian population, a model incorporating longitudinal EHR data^16^ predicted future suicidal behavior in both inpatient and outpatient visits with good sensitivity (33%-45%) and specificity (90%-95%). The Mental Health Research Network used data across 7 health systems, including the 9-item Patient Health Questionnaire (PHQ-9),^17^ to predict suicide attempt and death within 90 days, with the top 5% of risk scores accounting for 43% to 48% of suicide attempts. In a pediatric ED, a model combining EHR data and brief suicide screening (Ask Suicide Questionnaire [ASQ])^36^ outperformed screening alone in the prediction of subsequent suicide-related visits.

Integrating clinical and statistical risk predictions might provide more effective suicide risk detection.^18^ However, it remains to be established whether these alternative methods are “competing, complementary, or merely duplicative.”^18^ Vanderbilt University Medical Center (VUMC) has implemented universal screening in the ED with the C-SSRS since 2018. In parallel, our team has deployed the Vanderbilt Suicide Attempt and Ideation Likelihood (VSAIL) model, which has generated real-time suicide risk predictions across the enterprise since June 2019. In this study, we evaluated the ability of the C-SSRS alone to predict suicide attempt (SA) and suicidal ideation (SI) and compared this performance to our real-time suicide risk prediction model. Finally, we analyzed the performance of our risk model combined with the C-SSRS to evaluate whether synergistic effects improve performance.

## Methods

### Data Collection and Case Definition

We studied an observational cohort of adult patients (aged ≥18 years) at VUMC from June 2019 to September 2020, extracted from the Vanderbilt Research Derivative, a clinical research repository.^20^ We extracted C-SSRS response data from all screened patients from the Clarity Database in the EPIC Systems EHR. At each index visit in which the C-SSRS was administered, we also extracted the corresponding VSAIL risk scores generated at the start of each encounter. We included patient encounters in the inpatient (general medical and psychiatric), ambulatory surgical, and ED settings. The institutional review board at VUMC approved the analyses needed in this study. Because of the impracticality of obtaining patient consent in this EHR-based study, analyses were approved with waiver of consent. This study followed the Transparent Reporting of a Multivariable Prediction Model for Individual Prognosis or Diagnosis (TRIPOD) reporting guidelines.^35^

The primary outcomes in this study were SA and SI, defined as separate events by coded self-injurious thoughts and behaviors (SITBs) occurring within 7, 30, 60, 90, and 180 days after the discharge date of each documented visit during the time period. SITBs were extracted from encounter diagnosis documentation encoded as *International Classification of Diseases, Ninth Revision, Clinical Modification *(*ICD-9-CM*) and *International Statistical Classification of Diseases, Tenth Revision, Clinical Modification *(*ICD-10-CM*) stored in our EHR. To avoid circularity when using the C-SSRS to predict future SI and SA, we defined these outcomes exclusively through *ICD* codes without any reference to clinical screening. In eTable 1 in the Supplement, we provide a reference list of codes used for SI and SA from published sources.^21^

### Universal Screening at VUMC

VUMC implemented universal screening using the C-SSRS in June 2018 in line with Joint Commission mandates.^22^ The timing of screening during clinical workflows varied considerably between sites and according to competing clinical priorities. Screening was performed during triage for ED encounters and during every shift for patients admitted to the psychiatric hospital. In outpatient clinical settings, screening was performed sporadically as dictated by clinical need (eg, patient-reported symptoms prompting suicide risk screening). Registered nurses typically administered the C-SSRS to patients face to face, eg, at triage in the ED.

The brief version of the C-SSRS consists of 6 cascading questions related to suicidal thoughts and behaviors. The 6 components of the brief C-SSRS implemented at VUMC were: question (Q) 1 (wish to be dead), Q 2 (suicidal thoughts), Q 3 (suicidal thoughts with method without specific plan or intent), Q 4 (suicidal intent without specific plan), Q 5 (suicidal intent with specific plan), and Q 6 (suicidal behavior). All screened patients were asked Qs 1, 2, and 6. Qs 3 to 5 were only asked if patients answered yes to Q 2.

Each question was asked with respect to occurrences within the past month or since the last assessment. Since there are many permutations of answers, C-SSRS scores were aggregated into an ordered series of risk groups determined by the VUMC safety board: green, indicating no reported ideation or behavior; yellow, wish to be dead, suicidal thoughts without method or plan, or suicidal behavior more than 1 year ago; orange, suicidal thoughts with method but without plan or suicidal behavior between 3 months and 1 year ago; and red, suicidal intent with or without plan or suicidal behavior within 3 months. If patients scored yellow, orange, or red, interventions were recommended by automated clinician-focused advisories in the patient medical record. These C-SSRS risk classifications used at VUMC do not correspond directly to the scoring methods validated by Posner et al.^13^

### Real-time Suicide Risk Prediction Model

The VSAIL model was initially trained using the random forest algorithm on a heterogenous mix of adult VUMC patients that included 3250 manually validated SA cases and a set of 12 695 controls with no history of SA.^23^ The model uses historical EHR data, including the following features: demographic data (age, sex, race), diagnostic codes, medication data, past health care utilization, and area deprivation index by patient zip code. Full model training and validation details have been published elsewhere.^19,23^

Since its deployment in 2019 to our production EHR environment (Epic Systems Corporation), the VSAIL model has silently calculated predictions of suicide risk at the start of routine clinical visits. Clinical decision support prompted by VSAIL has been designed in parallel but was not active during the period of this research.^24^ Although the VSAIL model was initially trained to predict SA and SI at 30 days, it has been validated for SA and SI across a variety of time periods (eg, 7, 30, 60, 90, and 180 days).^23,25^ Therefore, for each index visit, we used the associated VSAIL score as the predicted risk of SA and SI occurring within each corresponding time interval. A comprehensive evaluation is provided for 30-day outcomes based on previous selection of this prediction target.

### Statistical Validity of the C-SSRS

The data were transformed to the encounter level, with each row representing a unique patient visit. Only the responses associated with the index visit were considered, and a patient’s historical screening responses were ignored. Patient responses to the 6 C-SSRS components were represented as binary features with yes represented as 1 and 0 otherwise. An unstructured text field containing screener comments was also transformed to a binary feature in which nonnull values were represented as 1 and null values as 0. Using these covariate features, we fit separate logistic regression models for SA and SI within each time period. We calculated 95% CIs for the odds ratio (OR) of each covariate and tested the statistical significance of the observed effect sizes.

To assess the predictive validity of the C-SSRS triage system used at VUMC to estimate risk of SA and SI, we developed a rule-based model that classified patients scoring red as high-risk (predicted class 1) and all others as low-risk (predicted class 0). To optimally compare and combine face-to-face screening assessments with VSAIL predictions, we also trained a logistic regression model using the C-SSRS component features and predicted suicide risk at each index visit in the cohort. We generated out-of-sample predictions for each observation by using 5-fold cross-validation and combining the hold out set predictions from each fold. Based on an initial performance comparison and the continuous output of the VSAIL model, we used the similarly formatted C-SSRS regression model predictions, rather than the binary output of the C-SSRS rule-based model, to develop ensemble models.

### Ensemble Learning and Model Evaluation

Since the VSAIL model was already implemented in production and calculated in real-time, we avoided retraining in favor of ensemble methods that combined the C-SSRS regression and VSAIL predictions. This scenario is an example of late fusion, which combines the results of models trained separately into a single model.^34^ We used the mean and the maximum of these 2 predictions to test simple aggregations. We also calculated a weighted average equal to the C-SSRS regression for medium- to high-risk categories (ie, yellow, orange, or red), and the VSAIL prediction for low-risk categories (ie, green). To accommodate safety concerns with real-time deployment, the weighted average favored the C-SSRS for patients with active suicidal thoughts and behaviors, while favoring historical EHR data otherwise. Finally, we trained a lasso regression model with 5-fold cross-validation that used both the C-SSRS regression and VSAIL predictions as input features and produced a final risk prediction. To evaluate the lasso model, we combined hold out set predictions from each cross-validation fold.

We assessed standard discrimination performance metrics: area under the receiver operating characteristic curve (AUROC), area under the precision-recall curve (AUPR), sensitivity, specificity, positive predictive value (PPV), and negative predictive (NPV). Since AUROC can be problematic in classification problems with high case imbalance, it was used only as a preliminary comparison between models.^26^ Instead, we focused our primary evaluation on PPV and sensitivity at various prediction cutoffs to provide more informative metrics for clinical decision-making and risk detection with rare outcomes. To assess the added value of ensemble models, we calculated the integrated discrimination improvement (IDI), a measure of the improvement in integrated sensitivity and specificity.^43^

### Statistical Analysis

Statistical analyses were conducted using Python version 3.8.2 (Python Software Foundation). Hypotheses tests were 2-sided and specified at a significance level of .05.

## Results

Our cohort included 120 398 unique index-visits for 83 394 patients (mean [SD] age, 51.2 [20.6] years; 38 107 [46%] men; 45 273 [54%] women; 13 644 [16%] Black; 63 869 [77%] White). SA was documented in 84 (0.07%), 205 (0.17%), 272 (0.23%), 356 (0.30%), and 514 (0.43%) cases at 7, 30, 60, 90, and 180 days, respectively. SI was documented in 614 (0.51%), 1486 (1.23%), 2036 (1.69%), 2433 (2.02%), 3126 (2.60%) cases at 7, 30, 60, 90, and 180 days, respectively (Table 1).

**Table 1.** Study Demographic Characteristics and Outcome Prevalence

Time period, d	Total (N = 120 398)	Sex			Race				Hospital setting				
		Female (n = 63 820)	Male (n = 56 563)	Unknown (n = 15)	Black (n = 21 542)	White (n = 91 315)	Other (n = 6138)^a^	Unknown (n = 1403)	ED (n = 35 312)	IP (n = 41 211)	Other (n = 8880)^b^	Psychiatric ED (n = 2413)	AS (n = 32 582)
**Suicide attempt**													
7	84 (0.07)	34 (0.05)	50 (0.09)	0	19 (0.09)	64 (0.07)	1 (0.02)	0	27 (0.08)	34 (0.08)	10 (0.11)	9 (0.37)	4 (0.01)
30	205 (0.17)	75 (0.12)	130 (0.23)	0	44 (0.20)	159 (0.17)	2 (0.03)	0	62 (0.18)	89 (0.22)	20 (0.23)	27 (1.12)	7 (0.02)
60	272 (0.23)	93 (0.15)	179 (0.32)	0	57 (0.26)	213 (0.23)	2 (0.03)	0	81 (0.23)	117 (0.28)	27 (0.3)	38 (1.57)	9 (0.03)
90	356 (0.30)	124 (0.19)	232 (0.41)	0	75 (0.35)	279 (0.31)	2 (0.03)	0	101 (0.29)	143 (0.35)	36 (0.41)	62 (2.57)	14 (0.04)
180	514 (0.43)	172 (0.27)	342 (0.60)	0	106 (0.49)	404 (0.44)	4 (0.07)	0	156 (0.44)	204 (0.50)	39 (0.44)	98 (4.06)	17 (0.05)
**Suicidal ideation**													
7	614 (0.51)	155 (0.24)	459 (0.81)	0	160 (0.74)	442 (0.48)	10 (0.16)	2 (0.14)	289 (0.82)	171 (0.41)	24 (0.27)	125 (5.18)	5 (0.02)
30	1486 (1.23)	399 (0.63)	1087 (1.92)	0	390 (1.81)	1066 (1.17)	27 (0.44)	3 (0.21)	620 (1.76)	494 (1.20)	61 (0.69)	296 (12.27)	15 (0.05)
60	2036 (1.69)	557 (0.87)	1479 (2.61)	0	527 (2.45)	1470 (1.61)	36 (0.59)	3 (0.21)	823 (2.33)	675 (1.64)	85 (0.96)	419 (17.36)	34 (0.1)
90	2433 (2.02)	707 (1.11)	1726 (3.05)	0	605 (2.81)	1776 (1.94)	49 (0.8)	3 (0.21)	981 (2.78)	808 (1.96)	103 (1.16)	490 (20.31)	51 (0.16)
180	3126 (2.60)	958 (1.50)	2168 (3.83)	0	766 (3.56)	2290 (2.51)	66 (1.08)	4 (0.29)	1269 (3.59)	1051 (2.55)	140 (1.58)	589 (24.41)	77 (0.24)

Abbreviations: AS, ambulatory surgery; ED, emergency department; IP, inpatient.

^a^ Other group includes Asian, American Indian or Alaskan Native, Native Hawaiian and other Pacific Islander, and those declining to answer.

^b^ Other includes observation visits, surgery admissions, and hospice.

### Logistic Regression With C-SSRS Response Features

**Figure 1.**
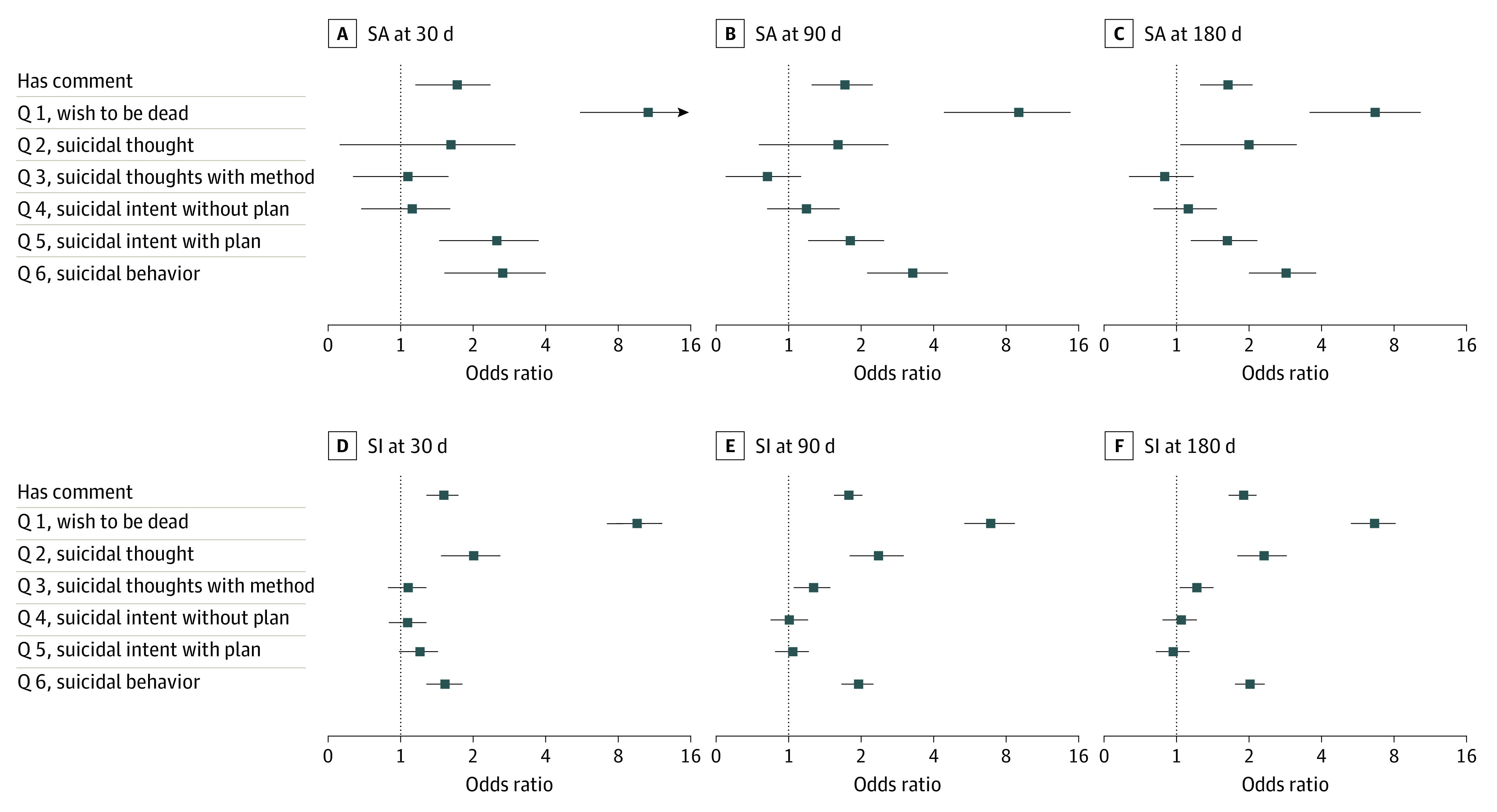
Summary of Logistic Regression With Columbia Suicide Severity Rating Scale (C-SSRS) Notes and Questions (Q) SA indicates suicide attempt; SI, suicidal ideation.

For SA and SI across all time intervals, positive responses to Q 1, Q 2, and Q 6 had the largest effect sizes and were most frequently statistically significant (Figure 1). For SA at 30 days, statistically significant C-SSRS components were Q 1 (OR, 8.80; 95% CI, 3.96-19.58), Q 5 (OR, 2.35; 95% CI, 1.45-3.82), and Q 6 (OR, 2.46; 95% CI, 1.51-4.03). For SI at 30 days, significant C-SSRS components were Q 1 (OR, 10.59; 95% CI, 7.98-14.04), Q 2 (OR, 2.04; 95% CI, 1.51-2.76), and Q 6 (OR, 1.56; 95% CI, 1.31-1.87). The full logistic regression results are included in eTable 2 in the Supplement.

### Area Under the Curve Metrics

**Figure 2.**
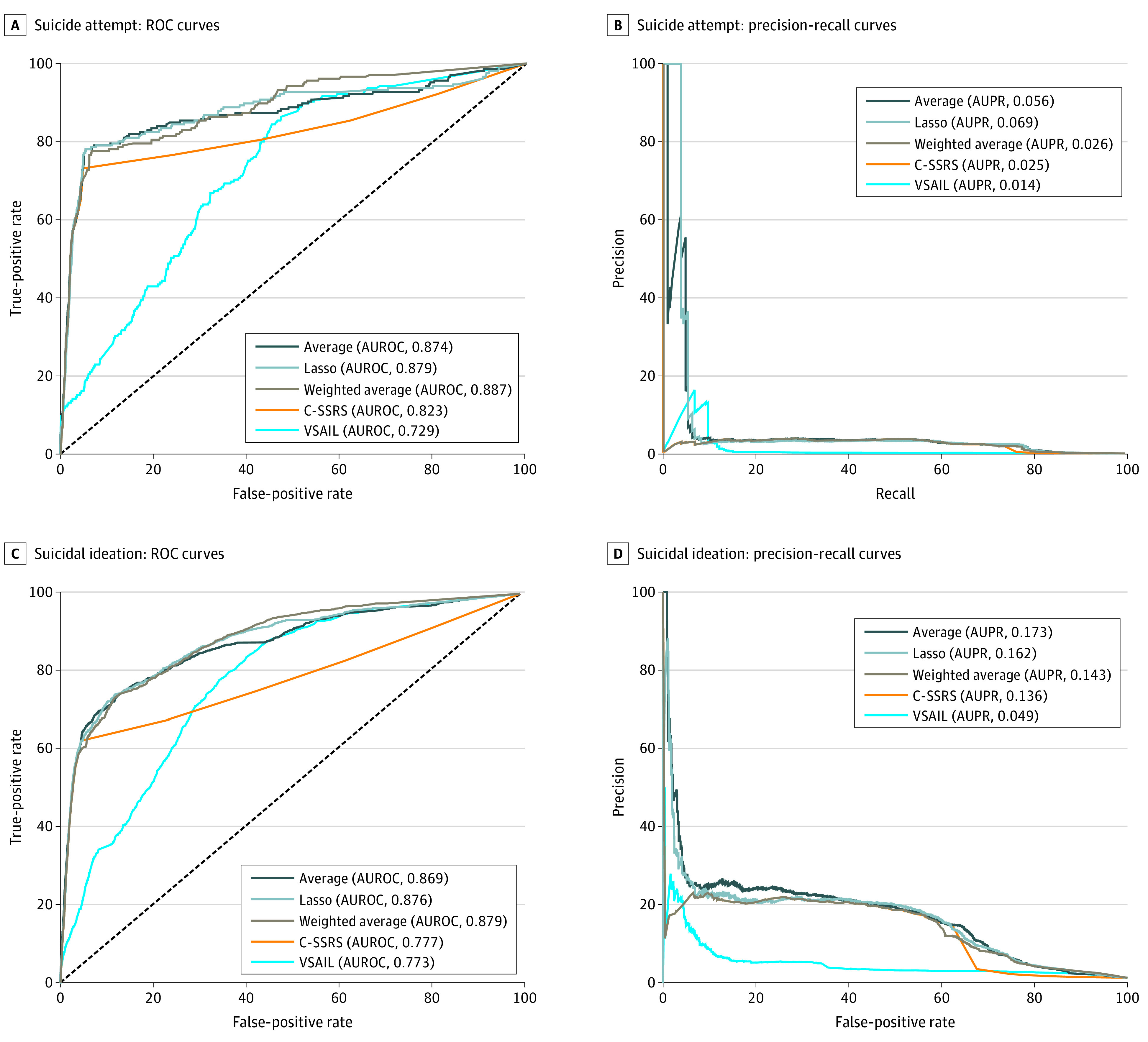
Receiver Operating Characteristic (ROC) and Precision-Recall Curves for Suicide Attempt and Suicidal Ideation at 30 Days AUPR indicates area under the precision-recall curve; AUROC, area under the receiver operating characteristic curve; C-SSRS, Columbia Suicide Severity Rating Scale; and VSAIL, Vanderbilt Suicide Attempt and Ideation Likelihood.

Across all time intervals, the logistic regression model trained with C-SSRS question-response features outperformed the C-SSRS triage system in terms of AUROC and AUPR. The combination of the C-SSRS regression and VSAIL models outperformed either alone in terms of AUROC and AUPR for both SA and SI at all time intervals. For example, within 30 days of an index visit, the combined models had an AUROC for SA of 0.874 to 0.887 and for SI of 0.869 to 0.879, while the VSAIL model had an AUROC for SA of 0.729 and for SI of 0.773 and the C-SSRS regression model had an AUROC for SA of 0.823 and for SI of 0.777. The weighted average had the highest AUROC among the ensemble models, while the unweighted average generally performed the best in terms of AUPR. The maximum prediction was equivalent to the C-SSRS regression and therefore disregarded in further evaluation. Both C-SSRS–based predictions outperformed the VSAIL model, except in terms of AUROC for longer-term SI. The VSAIL model showed equivalent or higher AUROC as time increased, while the AUROC for C-SSRS predictions decreased over time (Table 2 and Figure 2)

**Table 2.** AUROC and AUPR for VSAIL, C-SSRS, and Ensemble Models at All Time Intervals^a^

Model	Time following index visit, d									
	AUROC					AUPR				
	7	30	60	90	180	7	30	60	90	180
Suicide attempt										
VSAIL	0.726	0.728	0.729	0.733	0.756	0.005	0.014	0.015	0.016	0.021
C-SSRS red tier	0.804	0.757	0.738	0.737	0.710	0.012	0.021	0.025	0.032	0.037
C-SSRS regression	0.840	0.824	0.791	0.782	0.767	0.014	0.025	0.034	0.045	0.052
Unweighted average	0.873	0.877	0.860	0.858	0.870	0.051	0.054	0.055^b^	0.065^b^	0.074^b^
Maximum	0.840	0.824	0.791	0.782	0.767	0.014	0.025	0.034	0.045	0.052
Weighted average	0.907^b^	0.887^b^	0.884^b^	0.879^b^	0.877^b^	0.014	0.026	0.036	0.047	0.055
Lasso	0.869	0.880	0.871	0.869	0.874	0.073^b^	0.063^b^	0.055^b^	0.063	0.067
**Suicidal ideation**										
VSAIL	0.770	0.775	0.770	0.765	0.763	0.021	0.047	0.060	0.068	0.085
C-SSRS red tier	0.726	0.694	0.680	0.670	0.656	0.050	0.092	0.110	0.120	0.133
C-SSRS regression	0.814	0.783	0.754	0.743	0.728	0.072	0.139	0.170	0.190	0.210
Unweighted average	0.890	0.871	0.854	0.850	0.840	0.090^b^	0.173^b^	0.206^b^	0.223^b^	0.254^b^
Maximum	0.814	0.783	0.754	0.743	0.728	0.072	0.139	0.170	0.190	0.210
Weighted average	0.900^b^	0.880^b^	0.870^b^	0.862^b^	0.850^b^	0.075	0.145	0.181	0.204	0.228
Lasso	0.896	0.877	0.865	0.860	0.848	0.082	0.162	0.190	0.204	0.234

Abbreviations: AUPR, area under the precision-recall curve; AUROC, area under the receiver operating characteristic curve; C-SSRS, Columbia Suicide Severity Rating Scale; VSAIL, Vanderbilt Suicide Attempt and Ideation Likelihood.

^a^ Ensemble models combining the VSAIL and C-SSRS regression predictions include the unweighted average, maximum, weighted average, and lasso regression models.

^b^ Denotes the model with the best performance for each outcome by time period.

### Discrimination at 30 Days

At lower-risk cutoffs for outcomes occurring within 30 days, the VSAIL model had higher sensitivity and specificity than the C-SSRS regression model. At higher-risk thresholds, the C-SSRS regression model offered substantially higher PPV than the VSAIL model and continued to identify the majority of cases up until the 95th percentile. The ensemble models had higher sensitivity, specificity, and PPV than both the C-SSRS regression and VSAIL models across all risk thresholds, except for the top 1%, where the best ensemble model was equivalent to the C-SSRS regression. In the top 10% of SA risk, ensemble models had higher sensitivity (SA: 77.6%-79.5%, SI: 67.4%-70.1%) and PPV (SA: 1.3%-1.4%; SI: 8.3%-8.7%) than the C-SSRS regression (sensitivity for SA: 76.6%; sensitivity for SI: 68.8%; PPV for SA: 0.5%; PPV for SI: 3.5%) and VSAIL models (sensitivity for SA: 28.8%; sensitivity for SI: 35.1%; PPV for SA: 0.4%; PPV for SI: 3.9%). The red C-SSRS triage tier identified most SA cases (53.7%) with a PPV (3.8%) that rivaled the C-SSRS regression and ensemble models at the 99th risk percentile (Table 3). Full discrimination metrics by risk threshold are shown for all time periods in eTable 3 in the Supplement.

**Table 3.** Discrimination at Selected Risk Prediction Cutoffs for 30-Day Estimation

Risk cutoff	Risk threshold metrics, %			
	Specificity	Sensitivity	PPV	NPV
**Suicide attempt**				
C-SSRS triage				
Yellow	95.6	69.3	2.6	99.9
Orange	97.2	60.0	3.5	99.9
Red	97.7	53.7	3.8	99.9
C-SSRS regression, percentile				
>50	37.9	85.4	0.2	99.9
>75	56.8	82.0	0.3	99.9
>90	76.0	76.6	0.5	99.9
>95	95.1	73.2	2.5	100.0
>99	99.0	24.4	4.1	99.9
VSAIL, percentile				
>50	49.1	87.3	0.3	100.0
>75	75.0	50.2	0.3	99.9
>90	88.8	28.8	0.4	99.9
>95	95.0	17.1	0.6	99.9
>99	99.0	11.7	2.0	99.8
Ensemble, percentile				
>50	48.0-50.1	90.2-94.1	0.3-0.3	100.0-100.0
>75	75.0-75.1	82.0-84.4	0.6-0.6	100.0-100.0
>90	90.1-90.1	77.6-79.5	1.3-1.4	100.0-100.0
>95	95.0-95.1	70.2-75.6	2.3-2.6	99.9-100.0
>99	99.0-99.0	21.0-24.4	3.6-4.1	99.9-99.9
**Suicidal ideation**				
C-SSRS triage				
Yellow	96.1	58.5	16.0	99.5
Orange	97.6	46.2	19.7	99.3
Red	98.1	40.6	20.8	99.2
C-SSRS regression, percentile				
>50	38.2	83.3	1.7	99.5
>75	57.4	75.9	2.2	99.5
>90	76.5	68.8	3.5	99.5
>95	95.7	61.2	15.0	99.5
>99	99.2	17.4	20.9	99.0
VSAIL, percentile				
>50	49.6	90.9	2.2	99.8
>75	75.5	61.0	3.0	99.4
>90	89.0	35.1	3.9	99.1
>95	95.2	20.9	5.1	99.0
>99	99.1	7.5	9.3	98.8
Ensemble, percentile				
>50	45.6-50.5	90.9-95.2	2.1-2.3	99.8-99.9
>75	75.6-75.7	81.4-82.0	4.0-4.1	99.7-99.7
>90	90.7-90.8	67.4-70.1	8.3-8.7	99.6-99.6
>95	95.5-95.7	59.7-61.2	14.2-15.1	99.5-99.5
>99	99.2-99.2	17.0-19.7	20.9-24.3	99.0-99.0

Abbreviations: C-SSRS, Columbia Suicide Severity Rating Scale; NPV, negative predictive value; PPV, positive predictive value; VSAIL, Vanderbilt Suicide Attempt and Ideation Likelihood.

For SA occurring within 30 days, VSAIL had a higher AUPR (0.235) than the C-SSRS regression model (0.029) among psychiatric ED encounters. In the ED and inpatient settings, the C-SSRS regression had AUPR scores of 0.019 and 0.031, respectively, while the VSAIL model had AUPR values of 0.007 and 0.011, respectively. The VSAIL model performed better among White patients than patients with other racial or ethnic identities (AUPR: 0.107 vs 0.024), while the disparity was smaller for the C-SSRS regression (AUPR: 0.036 vs 0.024).

### Misclassification and Reclassification

For SA at 30 days, the IDI was 0.053 between the lasso and C-SSRS regression models (*P* < .001) and 0.510 between the lasso and VSAIL models (*P* < .001). For SI at 30 days, the IDI was 0.024 between the lasso and C-SSRS regression models (*P* < .001) and 0.462 between the lasso and VSAIL models (*P* < .001).

Of 514 SAs occurring within 180 days, the C-SSRS regression uniquely identified 237 cases within the highest-risk decile. The VSAIL model correctly stratified 73 cases that were not identified by the C-SSRS regression within the highest risk decile. Of the cases predicted by the C-SSRS alone, 220 (92.8%) had at least 1 prior visit with a diagnostic code for SA or SI, compared with 55 (75.3%) predicted by VSAIL alone. Cases identified by VSAIL alone, compared with the C-SSRS alone, were disproportionately male (61 [83.6%] vs 144 [60.8%]) and had a higher median (IQR) number of total visits (126 [78-302] vs 77 [21-137]) and years of EHR data (17.14 [3.97-26.85] vs 12.00 [2.05-19.54]).

## Discussion

We analyzed the predictive validity of the C-SSRS and evaluated the utility of combining face-to-face screening with an automated suicide risk prediction model. The primary finding was that the combination of the C-SSRS and VSAIL models outperformed either alone in the prediction of SA and SI at all time intervals. By leveraging the complementary strengths of historical EHR data and face-to-face screening, ensemble learning improved discrimination at various risk thresholds. In the highest risk decile for SA at 30 days, only the ensemble models surpassed thresholds (for PPV and sensitivity) required for suicide prediction models to deliver health economic benefit.^30^ We found this improvement (especially in PPV) to be clinically significant, although the costs and benefits of our ensemble approach will vary greatly between health care sites.

### Potential Explanations of Observed Synergy

While the sensitivity of the C-SSRS regression decreased over time and increased for the VSAIL model, ensemble models showed consistent performance across time. There was a distinct difference in the predictive time scale of the C-SSRS, which records dynamic, short-term indicators of a patient’s suicidal thoughts and behaviors, and the EHR-based model, which learns a patient’s underlying risk level by incorporating longitudinal clinical features. At the 50th risk percentile cutoff, the VSAIL model had higher sensitivity and PPV than the C-SSRS regression model for both SA and SI. At the 95th and 99th percentile cutoffs, the C-SSRS regression performed notably better. Ensemble models might have benefited from the relative strengths of the VSAIL and C-SSRS regression models at lower and higher risk thresholds, respectively. The C-SSRS predictions might have been limited by the commonality of patients denying SI despite being at high risk of SA and death.^27^ Performance of the VSAIL model may have suffered because some observations in the analysis did not have extensive historical clinical data. Ensemble methods seemed to mitigate the respective weaknesses of both data sources by exploiting their diversity while also synthesizing their independent, complementary strengths.

### Implications for Clinical Practice

Simon et al^17^ found suicide risk screening (patient self-report via the PHQ-9) to be an important predictive feature when included alongside other clinical variables in initial EHR model training. Our work builds on this by demonstrating that structured clinician assessment (ie, the C-SSRS) can be combined with existing risk prediction models for improved performance, even when screening data are not available at the time of EHR model training. Through our novel ensemble approach, we isolated and quantified the additive benefit of face-to-face screening when incorporated with EHR-based predictions. By also analyzing the discordance and potential synergies between these 2 existing risk prediction methods, we make novel contributions toward the reconciliation of statistical and clinical risk prediction, as outlined by Simon et al.^18^

A meta-analysis by Franklin et al^33^ showed poor predictive validity for screening instruments such as the C-SSRS. However, these instruments continue to be used clinically, and measuring their local predictive validity remains worthwhile. We found that the C-SSRS performs well locally, but its combination with automated scalable risk modeling performs better than either alone. Rapid assessments, such as the brief version of the C-SSRS used here or the ASQ, are likely preferable to longer assessments like the full C-SSRS (when used alone or in combination with EHR-based models).^37^

Clinical screening and EHR-based models have strengths and weaknesses beyond their predictive performance. In-person screening requires time, mental health resources (which are often limited), training on standardized assessments (eg, the C-SSRS), support from health care administrators, and workflow modifications.^37,40,41^ An important benefit of clinical screening is that it creates an opportunity for patient-physician dialogue that can lead to individualized treatment interventions. Although EHR-based machine learning can be automated at scale, developing, validating, and implementing predictive models requires a substantial initial resource investment. Ethical and legal issues around privacy, data usage, and accountability also hinder the adoption of machine learning in health care.^42^

Our findings highlight specific ways that face-to-face screening and EHR-based models might be used in tandem to overcome the challenges of predicting rare phenomena like SA and death. The low PPV currently offered by suicide risk models limits their potential utility in clinical practice, as falsely classifying patients as high-risk for suicide might worsen stigma and unnecessary interventions.^28,29^ Although this has been proposed elsewhere, our results empirically support using an EHR-based model as an initial detection mechanism that prompts further in-person screening.^16,19^ At the 50th risk percentile cutoff, the VSAIL model would have identified 18% to 35% more individuals with SA (25%-42% for ensemble models) that were not detected by C-SSRS triage. However, the higher PPV offered by the C-SSRS will likely be necessary to confidently recommend preventive care for individuals with high risk. Using EHR-based machine learning and face-to-face screening in this hierarchical series seems to leverage their complementary nature in a way that augments, rather than replaces, clinician-centered care.

Alternatively, it might be more natural in some settings (eg, those with universal screening) to use a series implementation that applies statistical prediction to patients screening negative. The C-SSRS outperformed the VSAIL model (especially in the short term), and SA cases identified by VSAIL alone were more likely to have no history of suicidal behavior (24.7% vs 7.2%). In settings where screening is widely administered, statistical prediction might be used secondarily to identify cases without prior suicidal behavior or with low screening risk due to nondisclosure. In-person screening and EHR-based models could also be (and often would be) implemented in parallel and combined with an ensemble model that outputs a final risk prediction and triggers clinical action. This would provide a marginally higher PPV than our C-SSRS triage system but would introduce many significant obstacles associated with using machine learning alone to dictate clinical interventions.^31^ Our results suggest that EHR-based models should incorporate available in-person screening data to improve sensitivity and PPV (especially at higher risk thresholds). For the majority of health care systems implementing face-to-face screening alone, incorporating EHR-based models can improve sensitivity at lower risk thresholds, provide continuous output for more specific decision cutoffs, and identify cases typically overlooked by clinician assessment (eg, instances of patient nondisclosure).

### Strengths and Future Work

A primary strength of this study is the collection of a large sample of EHR data with significant overlapping implementation periods for an automated prediction model and universal screening. Our cohort included a relatively large number of SA cases for an observational EHR-based study, which enabled us to develop and evaluate predictive risk models. This analysis greatly benefited from an existing prediction model developed on-site and supported by multiple validation studies.^19,23^ The C-SSRS is a validated screening tool that predicts suicidal behavior, which likely enabled us to demonstrate notable performance improvements when integrating face-to-face screening and an EHR-based model.^13^ SA and SI are more prevalent than suicide death, and targeting these outcomes might lead to more clinically useful risk models with acceptable PPV.^28^

Immediate extensions of this work include ongoing research using the VSAIL model to prompt suicide risk screening in a pragmatic clinical trial and concurrent efforts to incorporate the C-SSRS response data within our real-time modeling pipeline. Rigorous statistical analysis and replication are needed to further evaluate the discordance between statistical and clinical risk prediction across various patient cohorts. Our work leaves ample opportunity to explore alternate ways of integrating statistical and clinical risk predictions. Although lasso was chosen as a simple ensemble method, more complex algorithms, such as nonnegative least squares (NNLS), might improve performance.^38^ Additional attempts could allow clinicians to heuristically combine EHR-based predictions with in-person screening, in contrast to our ensemble learning approach. To validate the use of automated risk prediction and face-to-face screening in series, one must evaluate how screening methods would perform on the cohort initially identified by statistical risk prediction alone.

### Limitations

Limitations of this work include its confinement to a single medical center, with reliance on universal screening (potentially infeasible for some health systems) and a pretrained model, which may limit generalizability. Although we estimated that screening was administered for 98% of adult ED visits, our cohort excluded a larger set of patient visits (particularly those outside the ED) that did not include the screening. This underestimated the broader applicability of the VSAIL model and may have inflated PPV given the higher case prevalence in this cohort. Since patients with more than 1 visit were included multiple times, discrimination metrics might have been affected by the repeated-observations problem.^39^ By defining the outcomes of interest (SA or SI) based on *ICD* codes associated with follow-up visits at VUMC, we excluded cases occurring outside our health care system. *ICD* codes may have introduced diagnostic imprecision or excluded incompletely coded cases of suicide (eg, patients presenting in cardiac arrest or in overdose without obvious suicidal association).^32^ The *ICD-10-CM* codes used to define SA include language of “intentional self-harm” (eg, T36) or “suicide attempt” (T14). A caveat remains that intent to die cannot be gleaned from an *ICD-10-CM* code for “intentional self-harm” alone. Holistic ascertainment of suicide death was unavailable during the study period and therefore not considered as an outcome The C-SSRS was used to recommend preventive interventions, and this likely confounded the predictive performance of the C-SSRS model, as patients screening positive might have received treatments that prevented future suicidal events.

## Conclusions

In this study, ensemble models combining the C-SSRS and an EHR-based machine learning model outperformed either alone in the prediction of SA and SI. Differences across various risk thresholds, time periods, and characteristics of identified cases seem to underly a synergy between clinical and statistical risk prediction. The improvement (especially in PPV) from combining in-person screening and historical EHR data was clinically significant, although the costs and benefits of our ensemble approach will vary greatly between health care sites. Further research is needed to compare alternate ways of combining clinical and statistical risk prediction and to analyze the practical implications of implementing them in clinical systems.

## Notes

Supplement. **eTable 1. ***ICD-10-CM* and *ICD-9-CM* Codes for Suicide Attempt (SA) and Suicidal Ideation (SI) **eTable 2. **C-SSRS Logistic Regression Odds Ratios **eTable 3. **Discrimination Metrics by Risk Threshold Across All Time Periods

## References

[1] World Health Organization. Suicide in the world: global health estimates. Accessed September 20, 2021. https://apps.who.int/iris/handle/10665/326948

[2] Reger MA, Stanley IH, Joiner TE. Suicide mortality and coronavirus disease 2019—a perfect storm? JAMA Psychiatry. 2020;77(11):1093-1094. doi:10.1001/jamapsychiatry.2020.1060 32275300

[3] Olfson M, Blanco C, Wall M, . National trends in suicide attempts among adults in the United States. JAMA Psychiatry. 2017;74(11):1095-1103. doi:10.1001/jamapsychiatry.2017.2582 28903161 PMC5710225

[4] Boudreaux ED, Camargo CA Jr, Arias SA, . Improving suicide risk screening and detection in the emergency department. Am J Prev Med. 2016;50(4):445-453. doi:10.1016/j.amepre.2015.09.029 26654691 PMC4801719

[5] Doupnik SK, Rudd B, Schmutte T, . Association of suicide prevention interventions with subsequent suicide attempts, linkage to follow-up care, and depression symptoms for acute care settings: a systematic review and meta-analysis. JAMA Psychiatry. 2020;77(10):1021-1030. doi:10.1001/jamapsychiatry.2020.1586 32584936 PMC7301305

[6] Zalsman G, Hawton K, Wasserman D, . Suicide prevention strategies revisited: 10-year systematic review. Lancet Psychiatry. 2016;3(7):646-659. doi:10.1016/S2215-0366(16)30030-X 27289303

[7] Luoma JB, Martin CE, Pearson JL. Contact with mental health and primary care providers before suicide: a review of the evidence. Am J Psychiatry. 2002;159(6):909-916. doi:10.1176/appi.ajp.159.6.909 12042175 PMC5072576

[8] Rui P, Kang K. National Hospital Ambulatory Medical Care Survey: 2017 emergency department summary tables. National Center for Health Statistics. Accessed September 20, 2021. https://www.cdc.gov/nchs/data/nhamcs/web_tables/2017_ed_web_tables-508.pdf

[9] Pattyn E, Verhaeghe M, Sercu C, Bracke P. Public stigma and self-stigma: differential association with attitudes toward formal and informal help seeking. Psychiatr Serv. 2014;65(2):232-238. doi:10.1176/appi.ps.201200561 24233070

[10] Betz ME, Wintersteen M, Boudreaux ED, . Reducing suicide risk: challenges and opportunities in the emergency department. Ann Emerg Med. 2016;68(6):758-765. doi:10.1016/j.annemergmed.2016.05.030 27451339

[11] King CA, Horwitz A, Czyz E, Lindsay R. Suicide risk screening in healthcare settings: identifying males and females at risk. J Clin Psychol Med Settings. 2017;24(1):8-20. doi:10.1007/s10880-017-9486-y 28251427 PMC5439267

[12] The Columbia Lighthouse Project. About the protocol. Accessed September 20, 2021. https://cssrs.columbia.edu/the-columbia-scale-c-ssrs/about-the-scale/

[13] Posner K, Brown GK, Stanley B, . The Columbia-Suicide Severity Rating Scale: initial validity and internal consistency findings from three multisite studies with adolescents and adults. Am J Psychiatry. 2011;168(12):1266-1277. doi:10.1176/appi.ajp.2011.10111704 22193671 PMC3893686

[14] Sheehan DV, Alphs LD, Mao L, . Comparative validation of the S-STS, the ISST-Plus, and the C-SSRS for assessing the suicidal thinking and behavior FDA 2012 Suicidality Categories. Innov Clin Neurosci. 2014;11(9-10):32-46. 25520887 PMC4267798

[15] Kessler RC, Stein MB, Petukhova MV, ; Army STARRS Collaborators. Predicting suicides after outpatient mental health visits in the Army Study to Assess Risk and Resilience in Servicemembers (Army STARRS). Mol Psychiatry. 2017;22(4):544-551. doi:10.1038/mp.2016.110 27431294 PMC5247428

[16] Barak-Corren Y, Castro VM, Javitt S, . Predicting suicidal behavior from longitudinal electronic health records. Am J Psychiatry. 2017;174(2):154-162. doi:10.1176/appi.ajp.2016.16010077 27609239

[17] Simon GE, Johnson E, Lawrence JM, . Predicting suicide attempts and suicide deaths following outpatient visits using electronic health records. Am J Psychiatry. 2018;175(10):951-960. doi:10.1176/appi.ajp.2018.17101167 29792051 PMC6167136

[18] Simon GE, Matarazzo BB, Walsh CG, . Reconciling statistical and clinicians’ predictions of suicide risk. Psychiatr Serv. 2021;72(5):555-562. doi:10.1176/appi.ps.202000214 33691491 PMC10296777

[19] Walsh CG, Johnson KB, Ripperger M, . Prospective validation of an electronic health record-based, real-time suicide risk model. JAMA Netw Open. 2021;4(3):e211428. doi:10.1001/jamanetworkopen.2021.1428 33710291 PMC7955273

[20] Roden DM, Pulley JM, Basford MA, . Development of a large-scale de-identified DNA biobank to enable personalized medicine. Clin Pharmacol Ther. 2008;84(3):362-369. doi:10.1038/clpt.2008.89 18500243 PMC3763939

[21] Hedegaard H, Schoenbaum M, Claassen C, Crosby A, Holland K, Proescholdbell S. Issues in developing a surveillance case definition for nonfatal suicide attempt and intentional self-harm using *International Classification of Diseases, Tenth Revision, Clinical Modification* (*ICD-10-CM*) coded data. Natl Health Stat Report. 2018;(108):1-19. 29616901

[22] The Joint Commission. National patient safety goal for suicide prevention. November 2018. Accessed September 20, 2021. https://www.jointcommission.org/-/media/tjc/documents/standards/r3-reports/r3_18_suicide_prevention_hap_bhc_cah_11_4_19_final1.pdf

[23] Walsh CG, Ribeiro JD, Franklin JC. Predicting risk of suicide attempts over time through machine learning. Clin Psychol Sci. 2017;5(3):457-469. doi:10.1177/2167702617691560

[24] Lenert MC, Matheny ME, Walsh CG. Prognostic models will be victims of their own success, unless…. J Am Med Inform Assoc. 2019;26(12):1645-1650. doi:10.1093/jamia/ocz145 31504588 PMC6857506

[25] McKernan LC, Lenert MC, Crofford LJ, Walsh CG. Outpatient engagement and predicted risk of suicide attempts in fibromyalgia. Arthritis Care Res (Hoboken). 2019;71(9):1255-1263. doi:10.1002/acr.23748 30192068 PMC6405325

[26] Saito T, Rehmsmeier M. The precision-recall plot is more informative than the ROC plot when evaluating binary classifiers on imbalanced datasets. PLoS One. 2015;10(3):e0118432. doi:10.1371/journal.pone.0118432 25738806 PMC4349800

[27] Obegi JH. How common is recent denial of suicidal ideation among ideators, attempters, and suicide decedents? a literature review. Gen Hosp Psychiatry. 2021;72:92-95. doi:10.1016/j.genhosppsych.2021.07.009 34358807

[28] Belsher BE, Smolenski DJ, Pruitt LD, . Prediction models for suicide attempts and deaths: a systematic review and simulation. JAMA Psychiatry. 2019;76(6):642-651. doi:10.1001/jamapsychiatry.2019.0174 30865249

[29] Mulder R, Newton-Howes G, Coid JW. The futility of risk prediction in psychiatry. Br J Psychiatry. 2016;209(4):271-272. doi:10.1192/bjp.bp.116.184960 27698212

[30] Ross EL, Zuromski KL, Reis BY, Nock MK, Kessler RC, Smoller JW. Accuracy requirements for cost-effective suicide risk prediction among primary care patients in the US. JAMA Psychiatry. 2021;78(6):642-650. doi:10.1001/jamapsychiatry.2021.0089 33729432 PMC7970389

[31] Kelly CJ, Karthikesalingam A, Suleyman M, Corrado G, King D. Key challenges for delivering clinical impact with artificial intelligence. BMC Med. 2019;17(1):195. doi:10.1186/s12916-019-1426-2 31665002 PMC6821018

[32] Swain RS, Taylor LG, Braver ER, Liu W, Pinheiro SP, Mosholder AD. A systematic review of validated suicide outcome classification in observational studies. Int J Epidemiol. 2019;48(5):1636-1649. doi:10.1093/ije/dyz038 30907424

[33] Franklin JC, Ribeiro JD, Fox KR, . Risk factors for suicidal thoughts and behaviors: a meta-analysis of 50 years of research. Psychol Bull. 2017;143(2):187-232. doi:10.1037/bul0000084 27841450

[34] Gunes H, Piccardi M. Affect recognition from face and body: early fusion vs. late fusion. 2005 IEEE International Conference on Systems, Man and Cybernetics. Accessed April 6, 2022. https://ieeexplore.ieee.org/document/1571679 10.1109/TSMCB.2008.927269 19068431

[35] Collins GS, Reitsma JB, Altman DG, Moons KG. Transparent Reporting of a multivariable prediction model for Individual Prognosis Or Diagnosis (TRIPOD): the TRIPOD Statement. Br J Surg. 2015;102(3):148-158. doi:10.1002/bjs.9736 25627261

[36] Haroz EE, Kitchen C, Nestadt PS, Wilcox HC, DeVylder JE, Kharrazi H. Comparing the predictive value of suicide risk screening to the detection of suicide risk using electronic health records in an urban pediatric emergency department. medRxiv. Preprint posted online January 2, 2021. doi:10.1101/2020.12.27.20248829 PMC8961462 34515351

[37] Snyder DJ, Jordan BA, Aizvera J, . From pilot to practice: implementation of a suicide risk screening program in hospitalized medical patients. Jt Comm J Qual Patient Saf. 2020;46(7):417-426. doi:10.1016/j.jcjq.2020.04.011 32473966 PMC11173372

[38] LeDell E, van der Laan MJ, Petersen M. AUC-maximizing ensembles through metalearning. Int J Biostat. 2016;12(1):203-218. doi:10.1515/ijb-2015-0035 27227721 PMC4912128

[39] Michael H, Tian L, Ghebremichael M. The ROC curve for regularly measured longitudinal biomarkers. Biostatistics. 2019;20(3):433-451. doi:10.1093/biostatistics/kxy010 29608649 PMC6587928

[40] Labouliere CD, Vasan P, Kramer A, . “Zero Suicide”—a model for reducing suicide in United States behavioral healthcare. Suicidologi. 2018;23(1):22-30. doi:10.5617/suicidologi.6198 29970972 PMC6022755

[41] Nestadt PS, Triplett P, Mojtabai R, Berman AL. Universal screening may not prevent suicide. Gen Hosp Psychiatry. 2020;63:14-15. doi:10.1016/j.genhosppsych.2018.06.006 30072036 PMC7112159

[42] Vayena E, Blasimme A, Cohen IG. Machine learning in medicine: addressing ethical challenges. PLoS Med. 2018;15(11):e1002689. doi:10.1371/journal.pmed.1002689 30399149 PMC6219763

[43] Pencina MJ, D’Agostino RB Sr, D’Agostino RB Jr, Vasan RS. Evaluating the added predictive ability of a new marker: from area under the ROC curve to reclassification and beyond. Stat Med. 2008;27(2):157-172. doi:10.1002/sim.2929 17569110

