# Supplementary material for: Integration of Face-to-Face Screening With Real-time Machine Learning to Predict Risk of Suicide Among Adults
Source: JAMA Netw Open. 2022 May 13;5(5):e2212095. doi: 10.1001/jamanetworkopen.2022.12095 (PMC9107032; doi:10.1001/jamanetworkopen.2022.12095)
Supplement: Supplement. — eTable 1. ICD-10-CM and ICD-9-CM Codes for Suicide Attempt (SA) and Suicidal Ideation (SI) eTable 2. C-SSRS Logistic Regression Odds Ratios eTable 3. Discrimination Metrics by Risk Threshold Across All Time Periods [file jamanetwopen-e2212095-s001.pdf]

Supplemental Online Content

Wilimitis D, Turer RW, Ripperger M, et al. Integration of face-to-face screening with real-time machine learning to predict risk of suicide among adults. *JAMA Netw Open.* 2022;5(5):e2212095. doi:10.1001/jamanetworkopen.2022.12095

eTable 1. *ICD-10-CM* and *ICD-9-CM* Codes for Suicide Attempt (SA) and Suicidal Ideation (SI)

eTable 2. C-SSRS Logistic Regression Odds Ratios

eTable 3. Discrimination Metrics by Risk Threshold Across All Time Periods

This supplemental material has been provided by the authors to give readers additional information about their work.

© 2022 Wilimitis D et al. *JAMA Network Open.*

eTable 1. *ICD-10-CM* and *ICD-9-CM* Codes for Suicide Attempt (SA) and Suicidal Ideation (SI)

ICD CODE	CODE DESCRIPTION	ICD VERSION	OUTCOME
**E950.0**	Suicide and self-inflicted poisoning by analgesics, antipyretics, and antirheumatics	ICD9CM	SA
**E950.1**	Suicide and self-inflicted poisoning by barbiturates	ICD9CM	SA
**E950.2**	Suicide and self-inflicted poisoning by other sedatives and hypnotics	ICD9CM	SA
**E950.3**	Suicide and self-inflicted poisoning by tranquilizers and other psychotropic agents	ICD9CM	SA
**E950.4**	Suicide and self-inflicted poisoning by other specified drugs and medicinal substances	ICD9CM	SA
**E950.5**	Suicide and self-inflicted poisoning by unspecified drug or medicinal substance	ICD9CM	SA
**E950.6**	Suicide and self-inflicted poisoning by agricultural and horticultural chemical and pharmaceutical preparations other than plant foods and fertilizers	ICD9CM	SA
**E950.7**	Suicide and self-inflicted poisoning by corrosive and caustic substances	ICD9CM	SA
**E950.8**	Suicide and self-inflicted poisoning by arsenic and its compounds	ICD9CM	SA
**E950.9**	Suicide and self-inflicted poisoning by other and unspecified solid and liquid substances	ICD9CM	SA
**E951.0**	Suicide and self-inflicted poisoning by gas distributed by pipeline	ICD9CM	SA
**E951.1**	Suicide and self-inflicted poisoning by liquefied petroleum gas distributed in mobile containers	ICD9CM	SA
**E951.8**	Suicide and self-inflicted poisoning by other utility gas	ICD9CM	SA
**E952.0**	Suicide and self-inflicted poisoning by motor vehicle exhaust gas	ICD9CM	SA
**E952.1**	Suicide and self-inflicted poisoning by other carbon monoxide	ICD9CM	SA
**E952.8**	Suicide and self-inflicted poisoning by other specified gases and vapors	ICD9CM	SA
**E952.9**	Suicide and self-inflicted poisoning by unspecified gases and vapors	ICD9CM	SA
**E953.0**	Suicide and self-inflicted injury by hanging	ICD9CM	SA
**E953.1**	Suicide and self-inflicted injury by suffocation by plastic bag	ICD9CM	SA
**E953.8**	Suicide and self-inflicted injury by other specified means	ICD9CM	SA
**E953.9**	Suicide and self-inflicted injury by unspecified means	ICD9CM	SA
**E954**	Suicide and self-inflicted injury by submersion [drowning]	ICD9CM	SA
**E955.0**	Suicide and self-inflicted injury by handgun	ICD9CM	SA
**E955.1**	Suicide and self-inflicted injury by shotgun	ICD9CM	SA
**E955.2**	Suicide and self-inflicted injury by hunting rifle	ICD9CM	SA
**E955.3**	Suicide and self-inflicted injury by military firearms	ICD9CM	SA
**E955.4**	Suicide and self-inflicted injury by other and unspecified firearm	ICD9CM	SA
**E955.5**	Suicide and self-inflicted injury by explosives	ICD9CM	SA
**E955.6**	Suicide and self-inflicted injury by air gun	ICD9CM	SA
**E955.9**	Suicide and self-inflicted injury by firearms and explosives, unspecified	ICD9CM	SA
**E956**	Suicide and self-inflicted injury by cutting and piercing instrument	ICD9CM	SA
**E957.0**	Suicide and self-inflicted injuries by jumping from residential premises	ICD9CM	SA
**E957.1**	Suicide and self-inflicted injuries by jumping from other man-made structures	ICD9CM	SA
**E957.2**	Suicide and self-inflicted injuries by jumping from natural sites	ICD9CM	SA
**E957.9**	Suicide and self-inflicted injuries by jumping from unspecified site	ICD9CM	SA
**E958.0**	Suicide and self-inflicted injury by jumping or lying before moving object	ICD9CM	SA
**E958.1**	Suicide and self-inflicted injury by burns, fire	ICD9CM	SA

© 2022 Wilimitis D et al. *JAMA Network Open.*

**E958.2**	Suicide and self-inflicted injury by scald	ICD9CM	SA
**E958.3**	Suicide and self-inflicted injury by extremes of cold	ICD9CM	SA
**E958.4**	Suicide and self-inflicted injury by electrocution	ICD9CM	SA
**E958.5**	Suicide and self-inflicted injury by crashing of motor vehicle	ICD9CM	SA
**E958.6**	Suicide and self-inflicted injury by crashing of aircraft	ICD9CM	SA
**E958.7**	Suicide and self-inflicted injury by caustic substances, except poisoning	ICD9CM	SA
**E958.8**	Suicide and self-inflicted injury by other specified means	ICD9CM	SA
**E958.9**	Suicide and self-inflicted injury by unspecified means	ICD9CM	SA
**E959**	Late effects of self-inflicted injury	ICD9CM	SA
**T14.91**	Suicide attempt	ICD10CM	SA
**T14.91XA**	Suicide attempt, initial encounter	ICD10CM	SA
**T14.91XD**	Suicide attempt, subsequent encounter	ICD10CM	SA
**T14.91XS**	Suicide attempt, sequela	ICD10CM	SA
**T36.0X2A**	Poisoning by penicillins, intentional self-harm, initial encounter	ICD10CM	SA
**T36.0X2D**	Poisoning by penicillins, intentional self-harm, subsequent encounter	ICD10CM	SA
**T36.1X2A**	Poisoning by cephalosporins and other beta-lactam antibiotics, intentional self-harm, initial encounter	ICD10CM	SA
**T36.3X2A**	Poisoning by macrolides, intentional self-harm, initial encounter	ICD10CM	SA
**T36.4X2A**	Poisoning by tetracyclines, intentional self-harm, initial encounter	ICD10CM	SA
**T36.4X2D**	Poisoning by tetracyclines, intentional self-harm, subsequent encounter	ICD10CM	SA
**T36.8X2A**	Poisoning by other systemic antibiotics, intentional self-harm, initial encounter	ICD10CM	SA
**T36.92XA**	Poisoning by unspecified systemic antibiotic, intentional self-harm, initial encounter	ICD10CM	SA
**T37.0X2A**	Poisoning by sulfonamides, intentional self-harm, initial encounter	ICD10CM	SA
**T37.3X2A**	Poisoning by other antiprotozoal drugs, intentional self-harm, initial encounter	ICD10CM	SA
**T37.5X2A**	Poisoning by antiviral drugs, intentional self-harm, initial encounter	ICD10CM	SA
**T37.8X2A**	Poisoning by other specified systemic anti-infectives and antiparasitics, intentional self-harm, initial encounter	ICD10CM	SA
**T38.0X2A**	Poisoning by glucocorticoids and synthetic analogues, intentional self-harm, initial encounter	ICD10CM	SA
**T38.0X2D**	Poisoning by glucocorticoids and synthetic analogues, intentional self-harm, subsequent encounter	ICD10CM	SA
**T38.0X2S**	Poisoning by glucocorticoids and synthetic analogues, intentional self-harm, sequela	ICD10CM	SA
**T38.1X2A**	Poisoning by thyroid hormones and substitutes, intentional self-harm, initial encounter	ICD10CM	SA
**T38.1X2S**	Poisoning by thyroid hormones and substitutes, intentional self-harm, sequela	ICD10CM	SA
**T38.2X2A**	Poisoning by antithyroid drugs, intentional self-harm, initial encounter	ICD10CM	SA
**T38.3X2A**	Poisoning by insulin and oral hypoglycemic [antidiabetic] drugs, intentional self-harm, initial encounter	ICD10CM	SA
**T38.3X2D**	Poisoning by insulin and oral hypoglycemic [antidiabetic] drugs, intentional self-harm, subsequent encounter	ICD10CM	SA
**T38.3X2S**	Poisoning by insulin and oral hypoglycemic [antidiabetic] drugs, intentional self-harm, sequela	ICD10CM	SA
**T38.4X2A**	Poisoning by oral contraceptives, intentional self-harm, initial encounter	ICD10CM	SA
**T38.5X2A**	Poisoning by other estrogens and progestogens, intentional self-harm, initial encounter	ICD10CM	SA
**T38.5X2D**	Poisoning by other estrogens and progestogens, intentional self-harm, subsequent encounter	ICD10CM	SA
**T38.6X2A**	Poisoning by antigonadotrophins, antiestrogens, antiandrogens, not elsewhere classified, intentional self-harm, initial encounter	ICD10CM	SA
**T38.7X2A**	Poisoning by androgens and anabolic congeners, intentional self-harm, initial encounter	ICD10CM	SA

© 2022 Wilimitis D et al. *JAMA Network Open.*

**T38.802A**	Poisoning by unspecified hormones and synthetic substitutes, intentional self-harm, initial encounter	ICD10CM	SA
**T38.892A**	Poisoning by other hormones and synthetic substitutes, intentional self-harm, initial encounter	ICD10CM	SA
**T38.892D**	Poisoning by other hormones and synthetic substitutes, intentional self-harm, subsequent encounter	ICD10CM	SA
**T39.012A**	Poisoning by aspirin, intentional self-harm, initial encounter	ICD10CM	SA
**T39.012D**	Poisoning by aspirin, intentional self-harm, subsequent encounter	ICD10CM	SA
**T39.012S**	Poisoning by aspirin, intentional self-harm, sequela	ICD10CM	SA
**T39.092A**	Poisoning by salicylates, intentional self-harm, initial encounter	ICD10CM	SA
**T39.092D**	Poisoning by salicylates, intentional self-harm, subsequent encounter	ICD10CM	SA
**T39.1X2A**	Poisoning by 4-Aminophenol derivatives, intentional self-harm, initial encounter	ICD10CM	SA
**T39.1X2D**	Poisoning by 4-Aminophenol derivatives, intentional self-harm, subsequent encounter	ICD10CM	SA
**T39.1X2S**	Poisoning by 4-Aminophenol derivatives, intentional self-harm, sequela	ICD10CM	SA
**T39.2X2A**	Poisoning by pyrazolone derivatives, intentional self-harm, initial encounter	ICD10CM	SA
**T39.312A**	Poisoning by propionic acid derivatives, intentional self-harm, initial encounter	ICD10CM	SA
**T39.312D**	Poisoning by propionic acid derivatives, intentional self-harm, subsequent encounter	ICD10CM	SA
**T39.312S**	Poisoning by propionic acid derivatives, intentional self-harm, sequela	ICD10CM	SA
**T39.392A**	Poisoning by other nonsteroidal anti-inflammatory drugs [NSAID], intentional self-harm, initial encounter	ICD10CM	SA
**T39.392D**	Poisoning by other nonsteroidal anti-inflammatory drugs [NSAID], intentional self-harm, subsequent encounter	ICD10CM	SA
**T39.8X2A**	Poisoning by other nonopioid analgesics and antipyretics, not elsewhere classified, intentional self-harm, initial encounter	ICD10CM	SA
**T39.92XA**	Poisoning by unspecified nonopioid analgesic, antipyretic and antirheumatic, intentional self-harm, initial encounter	ICD10CM	SA
**T40.0X2A**	Poisoning by opium, intentional self-harm, initial encounter	ICD10CM	SA
**T40.1X2A**	Poisoning by heroin, intentional self-harm, initial encounter	ICD10CM	SA
**T40.1X2D**	Poisoning by heroin, intentional self-harm, subsequent encounter	ICD10CM	SA
**T40.1X2S**	Poisoning by heroin, intentional self-harm, sequela	ICD10CM	SA
**T40.2X2A**	Poisoning by other opioids, intentional self-harm, initial encounter	ICD10CM	SA
**T40.2X2D**	Poisoning by other opioids, intentional self-harm, subsequent encounter	ICD10CM	SA
**T40.2X2S**	Poisoning by other opioids, intentional self-harm, sequela	ICD10CM	SA
**T40.3X2A**	Poisoning by methadone, intentional self-harm, initial encounter	ICD10CM	SA
**T40.3X2D**	Poisoning by methadone, intentional self-harm, subsequent encounter	ICD10CM	SA
**T40.4X2A**	Poisoning by other synthetic narcotics, intentional self-harm, initial encounter	ICD10CM	SA
**T40.4X2D**	Poisoning by other synthetic narcotics, intentional self-harm, subsequent encounter	ICD10CM	SA
**T40.5X2A**	Poisoning by cocaine, intentional self-harm, initial encounter	ICD10CM	SA
**T40.5X2D**	Poisoning by cocaine, intentional self-harm, subsequent encounter	ICD10CM	SA
**T40.602A**	Poisoning by unspecified narcotics, intentional self-harm, initial encounter	ICD10CM	SA
**T40.602D**	Poisoning by unspecified narcotics, intentional self-harm, subsequent encounter	ICD10CM	SA
**T40.692A**	Poisoning by other narcotics, intentional self-harm, initial encounter	ICD10CM	SA
**T40.7X2A**	Poisoning by cannabis (derivatives), intentional self-harm, initial encounter	ICD10CM	SA
**T40.8X2A**	Poisoning by lysergide [LSD], intentional self-harm, initial encounter	ICD10CM	SA
**T40.8X2D**	Poisoning by lysergide [LSD], intentional self-harm, subsequent encounter	ICD10CM	SA
**T40.902A**	Poisoning by unspecified psychodysleptics [hallucinogens], intentional self-harm, initial encounter	ICD10CM	SA

**T40.992A**	Poisoning by other psychodysleptics [hallucinogens], intentional self-harm, initial encounter	ICD10CM	SA
**T41.202A**	Poisoning by unspecified general anesthetics, intentional self-harm, initial encounter	ICD10CM	SA
**T41.3X2A**	Poisoning by local anesthetics, intentional self-harm, initial encounter	ICD10CM	SA
**T42.0X2A**	Poisoning by hydantoin derivatives, intentional self-harm, initial encounter	ICD10CM	SA
**T42.1X2A**	Poisoning by iminostilbenes, intentional self-harm, initial encounter	ICD10CM	SA
**T42.1X2D**	Poisoning by iminostilbenes, intentional self-harm, subsequent encounter	ICD10CM	SA
**T42.1X2S**	Poisoning by iminostilbenes, intentional self-harm, sequela	ICD10CM	SA
**T42.3X2A**	Poisoning by barbiturates, intentional self-harm, initial encounter	ICD10CM	SA
**T42.4X2A**	Poisoning by benzodiazepines, intentional self-harm, initial encounter	ICD10CM	SA
**T42.4X2D**	Poisoning by benzodiazepines, intentional self-harm, subsequent encounter	ICD10CM	SA
**T42.5X2A**	Poisoning by mixed antiepileptics, intentional self-harm, initial encounter	ICD10CM	SA
**T42.6X2A**	Poisoning by other antiepileptic and sedative-hypnotic drugs, intentional self-harm, initial encounter	ICD10CM	SA
**T42.6X2D**	Poisoning by other antiepileptic and sedative-hypnotic drugs, intentional self-harm, subsequent encounter	ICD10CM	SA
**T42.72XA**	Poisoning by unspecified antiepileptic and sedative-hypnotic drugs, intentional self-harm, initial encounter	ICD10CM	SA
**T42.72XD**	Poisoning by unspecified antiepileptic and sedative-hypnotic drugs, intentional self-harm, subsequent encounter	ICD10CM	SA
**T42.72XS**	Poisoning by unspecified antiepileptic and sedative-hypnotic drugs, intentional self-harm, sequela	ICD10CM	SA
**T42.8X2A**	Poisoning by antiparkinsonism drugs and other central muscle-tone depressants, intentional self-harm, initial encounter	ICD10CM	SA
**T42.8X2D**	Poisoning by antiparkinsonism drugs and other central muscle-tone depressants, intentional self-harm, subsequent encounter	ICD10CM	SA
**T43.012A**	Poisoning by tricyclic antidepressants, intentional self-harm, initial encounter	ICD10CM	SA
**T43.012D**	Poisoning by tricyclic antidepressants, intentional self-harm, subsequent encounter	ICD10CM	SA
**T43.012S**	Poisoning by tricyclic antidepressants, intentional self-harm, sequela	ICD10CM	SA
**T43.022A**	Poisoning by tetracyclic antidepressants, intentional self-harm, initial encounter	ICD10CM	SA
**T43.022D**	Poisoning by tetracyclic antidepressants, intentional self-harm, subsequent encounter	ICD10CM	SA
**T43.1X2A**	Poisoning by monoamine-oxidase-inhibitor antidepressants, intentional self-harm, initial encounter	ICD10CM	SA
**T43.202A**	Poisoning by unspecified antidepressants, intentional self-harm, initial encounter	ICD10CM	SA
**T43.202S**	Poisoning by unspecified antidepressants, intentional self-harm, sequela	ICD10CM	SA
**T43.212A**	Poisoning by selective serotonin and norepinephrine reuptake inhibitors, intentional self-harm, initial encounter	ICD10CM	SA
**T43.212D**	Poisoning by selective serotonin and norepinephrine reuptake inhibitors, intentional self-harm, subsequent encounter	ICD10CM	SA
**T43.212S**	Poisoning by selective serotonin and norepinephrine reuptake inhibitors, intentional self-harm, sequela	ICD10CM	SA
**T43.222A**	Poisoning by selective serotonin reuptake inhibitors, intentional self-harm, initial encounter	ICD10CM	SA
**T43.222D**	Poisoning by selective serotonin reuptake inhibitors, intentional self-harm, subsequent encounter	ICD10CM	SA
**T43.222S**	Poisoning by selective serotonin reuptake inhibitors, intentional self-harm, sequela	ICD10CM	SA
**T43.292A**	Poisoning by other antidepressants, intentional self-harm, initial encounter	ICD10CM	SA
**T43.292D**	Poisoning by other antidepressants, intentional self-harm, subsequent encounter	ICD10CM	SA
**T43.292S**	Poisoning by other antidepressants, intentional self-harm, sequela	ICD10CM	SA
**T43.3X2A**	Poisoning by phenothiazine antipsychotics and neuroleptics, intentional self-harm, initial encounter	ICD10CM	SA

© 2022 Wilimitis D et al. *JAMA Network Open.*

**T43.3X2D**	Poisoning by phenothiazine antipsychotics and neuroleptics, intentional self-harm, subsequent encounter	ICD10CM	SA
**T43.4X2A**	Poisoning by butyrophenone and thiothixene neuroleptics, intentional self-harm, initial encounter	ICD10CM	SA
**T43.502A**	Poisoning by unspecified antipsychotics and neuroleptics, intentional self-harm, initial encounter	ICD10CM	SA
**T43.502S**	Poisoning by unspecified antipsychotics and neuroleptics, intentional self-harm, sequela	ICD10CM	SA
**T43.592A**	Poisoning by other antipsychotics and neuroleptics, intentional self-harm, initial encounter	ICD10CM	SA
**T43.592D**	Poisoning by other antipsychotics and neuroleptics, intentional self-harm, subsequent encounter	ICD10CM	SA
**T43.592S**	Poisoning by other antipsychotics and neuroleptics, intentional self-harm, sequela	ICD10CM	SA
**T43.602A**	Poisoning by unspecified psychostimulants, intentional self-harm, initial encounter	ICD10CM	SA
**T43.612A**	Poisoning by caffeine, intentional self-harm, initial encounter	ICD10CM	SA
**T43.612D**	Poisoning by caffeine, intentional self-harm, subsequent encounter	ICD10CM	SA
**T43.612S**	Poisoning by caffeine, intentional self-harm, sequela	ICD10CM	SA
**T43.622A**	Poisoning by amphetamines, intentional self-harm, initial encounter	ICD10CM	SA
**T43.622D**	Poisoning by amphetamines, intentional self-harm, subsequent encounter	ICD10CM	SA
**T43.622S**	Poisoning by amphetamines, intentional self-harm, sequela	ICD10CM	SA
**T43.632A**	Poisoning by methylphenidate, intentional self-harm, initial encounter	ICD10CM	SA
**T43.632D**	Poisoning by methylphenidate, intentional self-harm, subsequent encounter	ICD10CM	SA
**T43.692A**	Poisoning by other psychostimulants, intentional self-harm, initial encounter	ICD10CM	SA
**T43.8X2A**	Poisoning by other psychotropic drugs, intentional self-harm, initial encounter	ICD10CM	SA
**T43.8X2D**	Poisoning by other psychotropic drugs, intentional self-harm, subsequent encounter	ICD10CM	SA
**T43.92XA**	Poisoning by unspecified psychotropic drug, intentional self-harm, initial encounter	ICD10CM	SA
**T44.0X2A**	Poisoning by anticholinesterase agents, intentional self-harm, initial encounter	ICD10CM	SA
**T44.1X2A**	Poisoning by other parasympathomimetics [cholinergics], intentional self-harm, initial encounter	ICD10CM	SA
**T44.1X2D**	Poisoning by other parasympathomimetics [cholinergics], intentional self-harm, subsequent encounter	ICD10CM	SA
**T44.2X2A**	Poisoning by ganglionic blocking drugs, intentional self-harm, initial encounter	ICD10CM	SA
**T44.3X2A**	Poisoning by other parasympatholytics [anticholinergics and antimuscarinics] and spasmolytics, intentional self-harm, initial encounter	ICD10CM	SA
**T44.3X2D**	Poisoning by other parasympatholytics [anticholinergics and antimuscarinics] and spasmolytics, intentional self-harm, subsequent encounter	ICD10CM	SA
**T44.4X2A**	Poisoning by predominantly alpha-adrenoreceptor agonists, intentional self-harm, initial encounter	ICD10CM	SA
**T44.4X2D**	Poisoning by predominantly alpha-adrenoreceptor agonists, intentional self-harm, subsequent encounter	ICD10CM	SA
**T44.6X2A**	Poisoning by alpha-adrenoreceptor antagonists, intentional self-harm, initial encounter	ICD10CM	SA
**T44.6X2D**	Poisoning by alpha-adrenoreceptor antagonists, intentional self-harm, subsequent encounter	ICD10CM	SA
**T44.7X2A**	Poisoning by beta-adrenoreceptor antagonists, intentional self-harm, initial encounter	ICD10CM	SA
**T44.7X2D**	Poisoning by beta-adrenoreceptor antagonists, intentional self-harm, subsequent encounter	ICD10CM	SA
**T44.7X2S**	Poisoning by beta-adrenoreceptor antagonists, intentional self-harm, sequela	ICD10CM	SA
**T44.8X2A**	Poisoning by centrally-acting and adrenergic-neuron-blocking agents, intentional self-harm, initial encounter	ICD10CM	SA
**T44.902A**	Poisoning by unspecified drugs primarily affecting the autonomic nervous system, intentional self-harm, initial encounter	ICD10CM	SA
**T44.992A**	Poisoning by other drug primarily affecting the autonomic nervous system, intentional self-harm, initial encounter	ICD10CM	SA

**T44.992D**	Poisoning by other drug primarily affecting the autonomic nervous system, intentional self-harm, subsequent encounter	ICD10CM	SA
**T45.0X2A**	Poisoning by antiallergic and antiemetic drugs, intentional self-harm, initial encounter	ICD10CM	SA
**T45.0X2D**	Poisoning by antiallergic and antiemetic drugs, intentional self-harm, subsequent encounter	ICD10CM	SA
**T45.1X2A**	Poisoning by antineoplastic and immunosuppressive drugs, intentional self-harm, initial encounter	ICD10CM	SA
**T45.1X2D**	Poisoning by antineoplastic and immunosuppressive drugs, intentional self-harm, subsequent encounter	ICD10CM	SA
**T45.2X2A**	Poisoning by vitamins, intentional self-harm, initial encounter	ICD10CM	SA
**T45.2X2D**	Poisoning by vitamins, intentional self-harm, subsequent encounter	ICD10CM	SA
**T45.4X2A**	Poisoning by iron and its compounds, intentional self-harm, initial encounter	ICD10CM	SA
**T45.4X2D**	Poisoning by iron and its compounds, intentional self-harm, subsequent encounter	ICD10CM	SA
**T45.512A**	Poisoning by anticoagulants, intentional self-harm, initial encounter	ICD10CM	SA
**T45.512D**	Poisoning by anticoagulants, intentional self-harm, subsequent encounter	ICD10CM	SA
**T45.522A**	Poisoning by antithrombotic drugs, intentional self-harm, initial encounter	ICD10CM	SA
**T45.522D**	Poisoning by antithrombotic drugs, intentional self-harm, subsequent encounter	ICD10CM	SA
**T45.7X2A**	Poisoning by anticoagulant antagonists, vitamin K and other coagulants, intentional self-harm, initial encounter	ICD10CM	SA
**T45.8X2A**	Poisoning by other primarily systemic and hematological agents, intentional self-harm, initial encounter	ICD10CM	SA
**T46.0X2A**	Poisoning by cardiac-stimulant glycosides and drugs of similar action, intentional self-harm, initial encounter	ICD10CM	SA
**T46.1X2A**	Poisoning by calcium-channel blockers, intentional self-harm, initial encounter	ICD10CM	SA
**T46.1X2D**	Poisoning by calcium-channel blockers, intentional self-harm, subsequent encounter	ICD10CM	SA
**T46.2X2A**	Poisoning by other antidysrhythmic drugs, intentional self-harm, initial encounter	ICD10CM	SA
**T46.4X2A**	Poisoning by angiotensin-converting-enzyme inhibitors, intentional self-harm, initial encounter	ICD10CM	SA
**T46.4X2D**	Poisoning by angiotensin-converting-enzyme inhibitors, intentional self-harm, subsequent encounter	ICD10CM	SA
**T46.5X2A**	Poisoning by other antihypertensive drugs, intentional self-harm, initial encounter	ICD10CM	SA
**T46.5X2D**	Poisoning by other antihypertensive drugs, intentional self-harm, subsequent encounter	ICD10CM	SA
**T46.5X2S**	Poisoning by other antihypertensive drugs, intentional self-harm, sequela	ICD10CM	SA
**T46.6X2A**	Poisoning by antihyperlipidemic and antiarteriosclerotic drugs, intentional self-harm, initial encounter	ICD10CM	SA
**T46.7X2A**	Poisoning by peripheral vasodilators, intentional self-harm, initial encounter	ICD10CM	SA
**T46.7X2D**	Poisoning by peripheral vasodilators, intentional self-harm, subsequent encounter	ICD10CM	SA
**T46.8X2A**	Poisoning by antivaricose drugs, including sclerosing agents, intentional self-harm, initial encounter	ICD10CM	SA
**T47.0X2A**	Poisoning by histamine H2-receptor blockers, intentional self-harm, initial encounter	ICD10CM	SA
**T47.0X2D**	Poisoning by histamine H2-receptor blockers, intentional self-harm, subsequent encounter	ICD10CM	SA
**T47.1X2A**	Poisoning by other antacids and anti-gastric-secretion drugs, intentional self-harm, initial encounter	ICD10CM	SA
**T47.1X2D**	Poisoning by other antacids and anti-gastric-secretion drugs, intentional self-harm, subsequent encounter	ICD10CM	SA
**T47.3X2A**	Poisoning by saline and osmotic laxatives, intentional self-harm, initial encounter	ICD10CM	SA
**T47.4X2A**	Poisoning by other laxatives, intentional self-harm, initial encounter	ICD10CM	SA
**T47.4X2D**	Poisoning by other laxatives, intentional self-harm, subsequent encounter	ICD10CM	SA
**T47.6X2A**	Poisoning by antidiarrheal drugs, intentional self-harm, initial encounter	ICD10CM	SA
**T47.6X2D**	Poisoning by antidiarrheal drugs, intentional self-harm, subsequent encounter	ICD10CM	SA

© 2022 Wilimitis D et al. *JAMA Network Open.*

**T47.92XA**	Poisoning by unspecified agents primarily affecting the gastrointestinal system, intentional self-harm, initial encounter	ICD10CM	SA
**T48.1X2A**	Poisoning by skeletal muscle relaxants [neuromuscular blocking agents], intentional self-harm, initial encounter	ICD10CM	SA
**T48.1X2D**	Poisoning by skeletal muscle relaxants [neuromuscular blocking agents], intentional self-harm, subsequent encounter	ICD10CM	SA
**T48.1X2S**	Poisoning by skeletal muscle relaxants [neuromuscular blocking agents], intentional self-harm, sequela	ICD10CM	SA
**T48.202A**	Poisoning by unspecified drugs acting on muscles, intentional self-harm, initial encounter	ICD10CM	SA
**T48.3X2A**	Poisoning by antitussives, intentional self-harm, initial encounter	ICD10CM	SA
**T48.3X2D**	Poisoning by antitussives, intentional self-harm, subsequent encounter	ICD10CM	SA
**T48.4X2A**	Poisoning by expectorants, intentional self-harm, initial encounter	ICD10CM	SA
**T48.4X2D**	Poisoning by expectorants, intentional self-harm, subsequent encounter	ICD10CM	SA
**T48.5X2A**	Poisoning by other anti-common-cold drugs, intentional self-harm, initial encounter	ICD10CM	SA
**T48.6X2A**	Poisoning by antiasthmatics, intentional self-harm, initial encounter	ICD10CM	SA
**T48.6X2D**	Poisoning by antiasthmatics, intentional self-harm, subsequent encounter	ICD10CM	SA
**T49.0X2A**	Poisoning by local antifungal, anti-infective and anti-inflammatory drugs, intentional self-harm, initial encounter	ICD10CM	SA
**T49.0X2S**	Poisoning by local antifungal, anti-infective and anti-inflammatory drugs, intentional self-harm, sequela	ICD10CM	SA
**T49.1X2A**	Poisoning by antipruritics, intentional self-harm, initial encounter	ICD10CM	SA
**T49.1X2D**	Poisoning by antipruritics, intentional self-harm, subsequent encounter	ICD10CM	SA
**T49.2X2A**	Poisoning by local astringents and local detergents, intentional self-harm, initial encounter	ICD10CM	SA
**T49.3X2A**	Poisoning by emollients, demulcents and protectants, intentional self-harm, initial encounter	ICD10CM	SA
**T49.4X2A**	Poisoning by keratolytics, keratoplastics, and other hair treatment drugs and preparations, intentional self-harm, initial encounter	ICD10CM	SA
**T49.6X2A**	Poisoning by otorhinolaryngological drugs and preparations, intentional self-harm, initial encounter	ICD10CM	SA
**T49.6X2D**	Poisoning by otorhinolaryngological drugs and preparations, intentional self-harm, subsequent encounter	ICD10CM	SA
**T49.8X2A**	Poisoning by other topical agents, intentional self-harm, initial encounter	ICD10CM	SA
**T49.92XA**	Poisoning by unspecified topical agent, intentional self-harm, initial encounter	ICD10CM	SA
**T49.92XD**	Poisoning by unspecified topical agent, intentional self-harm, subsequent encounter	ICD10CM	SA
**T50.0X2A**	Poisoning by mineralocorticoids and their antagonists, intentional self-harm, initial encounter	ICD10CM	SA
**T50.1X2A**	Poisoning by loop [high-ceiling] diuretics, intentional self-harm, initial encounter	ICD10CM	SA
**T50.2X2A**	Poisoning by carbonic-anhydrase inhibitors, benzothiadiazides and other diuretics, intentional self-harm, initial encounter	ICD10CM	SA
**T50.2X2D**	Poisoning by carbonic-anhydrase inhibitors, benzothiadiazides and other diuretics, intentional self-harm, subsequent encounter	ICD10CM	SA
**T50.3X2A**	Poisoning by electrolytic, caloric and water-balance agents, intentional self-harm, initial encounter	ICD10CM	SA
**T50.3X2D**	Poisoning by electrolytic, caloric and water-balance agents, intentional self-harm, subsequent encounter	ICD10CM	SA
**T50.4X2A**	Poisoning by drugs affecting uric acid metabolism, intentional self-harm, initial encounter	ICD10CM	SA
**T50.5X2A**	Poisoning by appetite depressants, intentional self-harm, initial encounter	ICD10CM	SA
**T50.6X2A**	Poisoning by antidotes and chelating agents, intentional self-harm, initial encounter	ICD10CM	SA
**T50.7X2A**	Poisoning by analeptics and opioid receptor antagonists, intentional self-harm, initial encounter	ICD10CM	SA
**T50.8X2A**	Poisoning by diagnostic agents, intentional self-harm, initial encounter	ICD10CM	SA

© 2022 Wilimitis D et al. *JAMA Network Open.*

**T50.902A**	Poisoning by unspecified drugs, medicaments and biological substances, intentional self-harm, initial encounter	ICD10CM	SA
**T50.902D**	Poisoning by unspecified drugs, medicaments and biological substances, intentional self-harm, subsequent encounter	ICD10CM	SA
**T50.902S**	Poisoning by unspecified drugs, medicaments and biological substances, intentional self-harm, sequela	ICD10CM	SA
**T50.992A**	Poisoning by other drugs, medicaments and biological substances, intentional self-harm, initial encounter	ICD10CM	SA
**T50.992D**	Poisoning by other drugs, medicaments and biological substances, intentional self-harm, subsequent encounter	ICD10CM	SA
**T50.992S**	Poisoning by other drugs, medicaments and biological substances, intentional self-harm, sequela	ICD10CM	SA
**T51.0X2A**	Toxic effect of ethanol, intentional self-harm, initial encounter	ICD10CM	SA
**T51.0X2D**	Toxic effect of ethanol, intentional self-harm, subsequent encounter	ICD10CM	SA
**T51.1X2A**	Toxic effect of methanol, intentional self-harm, initial encounter	ICD10CM	SA
**T51.2X2A**	Toxic effect of 2-Propanol, intentional self-harm, initial encounter	ICD10CM	SA
**T51.2X2D**	Toxic effect of 2-Propanol, intentional self-harm, subsequent encounter	ICD10CM	SA
**T51.3X2A**	Toxic effect of fusel oil, intentional self-harm, initial encounter	ICD10CM	SA
**T51.3X2D**	Toxic effect of fusel oil, intentional self-harm, subsequent encounter	ICD10CM	SA
**T51.8X2A**	Toxic effect of other alcohols, intentional self-harm, initial encounter	ICD10CM	SA
**T51.92XA**	Toxic effect of unspecified alcohol, intentional self-harm, initial encounter	ICD10CM	SA
**T51.92XD**	Toxic effect of unspecified alcohol, intentional self-harm, subsequent encounter	ICD10CM	SA
**T52.0X2A**	Toxic effect of petroleum products, intentional self-harm, initial encounter	ICD10CM	SA
**T52.3X2A**	Toxic effect of glycols, intentional self-harm, initial encounter	ICD10CM	SA
**T52.8X2A**	Toxic effect of other organic solvents, intentional self-harm, initial encounter	ICD10CM	SA
**T52.8X2D**	Toxic effect of other organic solvents, intentional self-harm, subsequent encounter	ICD10CM	SA
**T52.92XA**	Toxic effect of unspecified organic solvent, intentional self-harm, initial encounter	ICD10CM	SA
**T52.92XD**	Toxic effect of unspecified organic solvent, intentional self-harm, subsequent encounter	ICD10CM	SA
**T54.1X2A**	Toxic effect of other corrosive organic compounds, intentional self-harm, initial encounter	ICD10CM	SA
**T54.2X2A**	Toxic effect of corrosive acids and acid-like substances, intentional self-harm, initial encounter	ICD10CM	SA
**T54.2X2D**	Toxic effect of corrosive acids and acid-like substances, intentional self-harm, subsequent encounter	ICD10CM	SA
**T54.3X2A**	Toxic effect of corrosive alkalis and alkali-like substances, intentional self-harm, initial encounter	ICD10CM	SA
**T54.3X2D**	Toxic effect of corrosive alkalis and alkali-like substances, intentional self-harm, subsequent encounter	ICD10CM	SA
**T54.3X2S**	Toxic effect of corrosive alkalis and alkali-like substances, intentional self-harm, sequela	ICD10CM	SA
**T54.92XA**	Toxic effect of unspecified corrosive substance, intentional self-harm, initial encounter	ICD10CM	SA
**T54.92XD**	Toxic effect of unspecified corrosive substance, intentional self-harm, subsequent encounter	ICD10CM	SA
**T54.92XS**	Toxic effect of unspecified corrosive substance, intentional self-harm, sequela	ICD10CM	SA
**T55.0X2A**	Toxic effect of soaps, intentional self-harm, initial encounter	ICD10CM	SA
**T55.0X2D**	Toxic effect of soaps, intentional self-harm, subsequent encounter	ICD10CM	SA
**T55.1X2A**	Toxic effect of detergents, intentional self-harm, initial encounter	ICD10CM	SA
**T56.0X2A**	Toxic effect of lead and its compounds, intentional self-harm, initial encounter	ICD10CM	SA
**T56.4X2A**	Toxic effect of copper and its compounds, intentional self-harm, initial encounter	ICD10CM	SA
**T56.892A**	Toxic effect of other metals, intentional self-harm, initial encounter	ICD10CM	SA
**T56.892D**	Toxic effect of other metals, intentional self-harm, subsequent encounter	ICD10CM	SA
**T58.02XA**	Toxic effect of carbon monoxide from motor vehicle exhaust, intentional self-harm, initial encounter	ICD10CM	SA

© 2022 Wilimitis D et al. *JAMA Network Open.*

**T58.02XS**	Toxic effect of carbon monoxide from motor vehicle exhaust, intentional self-harm, sequela	ICD10CM	SA
**T58.12XA**	Toxic effect of carbon monoxide from utility gas, intentional self-harm, initial encounter	ICD10CM	SA
**T58.2X2A**	Toxic effect of carbon monoxide from incomplete combustion of other domestic fuels, intentional self-harm, initial encounter	ICD10CM	SA
**T58.2X2D**	Toxic effect of carbon monoxide from incomplete combustion of other domestic fuels, intentional self-harm, subsequent encounter	ICD10CM	SA
**T58.8X2A**	Toxic effect of carbon monoxide from other source, intentional self-harm, initial encounter	ICD10CM	SA
**T58.8X2D**	Toxic effect of carbon monoxide from other source, intentional self-harm, subsequent encounter	ICD10CM	SA
**T58.92XA**	Toxic effect of carbon monoxide from unspecified source, intentional self-harm, initial encounter	ICD10CM	SA
**T58.92XD**	Toxic effect of carbon monoxide from unspecified source, intentional self-harm, subsequent encounter	ICD10CM	SA
**T59.7X2A**	Toxic effect of carbon dioxide, intentional self-harm, initial encounter	ICD10CM	SA
**T59.812A**	Toxic effect of smoke, intentional self-harm, initial encounter	ICD10CM	SA
**T59.892A**	Toxic effect of other specified gases, fumes and vapors, intentional self-harm, initial encounter	ICD10CM	SA
**T59.92XA**	Toxic effect of unspecified gases, fumes and vapors, intentional self-harm, initial encounter	ICD10CM	SA
**T60.0X2A**	Toxic effect of organophosphate and carbamate insecticides, intentional self-harm, initial encounter	ICD10CM	SA
**T60.1X2A**	Toxic effect of halogenated insecticides, intentional self-harm, initial encounter	ICD10CM	SA
**T60.1X2D**	Toxic effect of halogenated insecticides, intentional self-harm, subsequent encounter	ICD10CM	SA
**T60.3X2A**	Toxic effect of herbicides and fungicides, intentional self-harm, initial encounter	ICD10CM	SA
**T60.4X2A**	Toxic effect of rodenticides, intentional self-harm, initial encounter	ICD10CM	SA
**T60.8X2A**	Toxic effect of other pesticides, intentional self-harm, initial encounter	ICD10CM	SA
**T60.92XA**	Toxic effect of unspecified pesticide, intentional self-harm, initial encounter	ICD10CM	SA
**T61.12XA**	Scombroid fish poisoning, intentional self-harm, initial encounter	ICD10CM	SA
**T62.0X2A**	Toxic effect of ingested mushrooms, intentional self-harm, initial encounter	ICD10CM	SA
**T62.2X2A**	Toxic effect of other ingested (parts of) plant(s), intentional self-harm, initial encounter	ICD10CM	SA
**T62.8X2A**	Toxic effect of other specified noxious substances eaten as food, intentional self-harm, initial encounter	ICD10CM	SA
**T63.302A**	Toxic effect of unspecified spider venom, intentional self-harm, initial encounter	ICD10CM	SA
**T63.332D**	Toxic effect of venom of brown recluse spider, intentional self-harm, subsequent encounter	ICD10CM	SA
**T63.462A**	Toxic effect of venom of wasps, intentional self-harm, initial encounter	ICD10CM	SA
**T63.482A**	Toxic effect of venom of other arthropod, intentional self-harm, initial encounter	ICD10CM	SA
**T63.92XA**	Toxic effect of contact with unspecified venomous animal, intentional self-harm, initial encounter	ICD10CM	SA
**T65.0X2A**	Toxic effect of cyanides, intentional self-harm, initial encounter	ICD10CM	SA
**T65.222A**	Toxic effect of tobacco cigarettes, intentional self-harm, initial encounter	ICD10CM	SA
**T65.292A**	Toxic effect of other tobacco and nicotine, intentional self-harm, initial encounter	ICD10CM	SA
**T65.292D**	Toxic effect of other tobacco and nicotine, intentional self-harm, subsequent encounter	ICD10CM	SA
**T65.292S**	Toxic effect of other tobacco and nicotine, intentional self-harm, sequela	ICD10CM	SA
**T65.892A**	Toxic effect of other specified substances, intentional self-harm, initial encounter	ICD10CM	SA
**T65.892D**	Toxic effect of other specified substances, intentional self-harm, subsequent encounter	ICD10CM	SA
**T65.892S**	Toxic effect of other specified substances, intentional self-harm, sequela	ICD10CM	SA
**T65.92XA**	Toxic effect of unspecified substance, intentional self-harm, initial encounter	ICD10CM	SA
**T65.92XD**	Toxic effect of unspecified substance, intentional self-harm, subsequent encounter	ICD10CM	SA
**T65.92XS**	Toxic effect of unspecified substance, intentional self-harm, sequela	ICD10CM	SA
**T71.132A**	Asphyxiation due to being trapped in bed linens, intentional self-harm, initial encounter	ICD10CM	SA

© 2022 Wilimitis D et al. *JAMA Network Open.*

**T71.132D**	Asphyxiation due to being trapped in bed linens, intentional self-harm, subsequent encounter	ICD10CM	SA
**T71.162A**	Asphyxiation due to hanging, intentional self-harm, initial encounter	ICD10CM	SA
**T71.162D**	Asphyxiation due to hanging, intentional self-harm, subsequent encounter	ICD10CM	SA
**T71.162S**	Asphyxiation due to hanging, intentional self-harm, sequela	ICD10CM	SA
**T71.192A**	Asphyxiation due to mechanical threat to breathing due to other causes, intentional self-harm, initial encounter	ICD10CM	SA
**X71.0XXA**	Intentional self-harm by drowning and submersion while in bathtub, initial encounter	ICD10CM	SA
**X71.0XXD**	Intentional self-harm by drowning and submersion while in bathtub, subsequent encounter	ICD10CM	SA
**X71.1XXA**	Intentional self-harm by drowning and submersion while in swimming pool, initial encounter	ICD10CM	SA
**X71.3XXA**	Intentional self-harm by drowning and submersion in natural water, initial encounter	ICD10CM	SA
**X71.8XXA**	Other intentional self-harm by drowning and submersion, initial encounter	ICD10CM	SA
**X71.9XXA**	Intentional self-harm by drowning and submersion, unspecified, initial encounter	ICD10CM	SA
**X72.XXXA**	Intentional self-harm by handgun discharge, initial encounter	ICD10CM	SA
**X72.XXXD**	Intentional self-harm by handgun discharge, subsequent encounter	ICD10CM	SA
**X72.XXXS**	Intentional self-harm by handgun discharge, sequela	ICD10CM	SA
**X73.0XXA**	Intentional self-harm by shotgun discharge, initial encounter	ICD10CM	SA
**X73.0XXD**	Intentional self-harm by shotgun discharge, subsequent encounter	ICD10CM	SA
**X73.1XXA**	Intentional self-harm by hunting rifle discharge, initial encounter	ICD10CM	SA
**X73.1XXD**	Intentional self-harm by hunting rifle discharge, subsequent encounter	ICD10CM	SA
**X73.1XXS**	Intentional self-harm by hunting rifle discharge, sequela	ICD10CM	SA
**X73.9XXS**	Intentional self-harm by unspecified larger firearm discharge, sequela	ICD10CM	SA
**X74.01XA**	Intentional self-harm by airgun, initial encounter	ICD10CM	SA
**X74.01XS**	Intentional self-harm by airgun, sequela	ICD10CM	SA
**X74.8XXA**	Intentional self-harm by other firearm discharge, initial encounter	ICD10CM	SA
**X74.8XXD**	Intentional self-harm by other firearm discharge, subsequent encounter	ICD10CM	SA
**X74.8XXS**	Intentional self-harm by other firearm discharge, sequela	ICD10CM	SA
**X74.9XXA**	Intentional self-harm by unspecified firearm discharge, initial encounter	ICD10CM	SA
**X74.9XXD**	Intentional self-harm by unspecified firearm discharge, subsequent encounter	ICD10CM	SA
**X74.9XXS**	Intentional self-harm by unspecified firearm discharge, sequela	ICD10CM	SA
**X75.XXXA**	Intentional self-harm by explosive material, initial encounter	ICD10CM	SA
**X75.XXXS**	Intentional self-harm by explosive material, sequela	ICD10CM	SA
**X76**	Intentional self-harm by smoke, fire and flames	ICD10CM	SA
**X76.XXXA**	Intentional self-harm by smoke, fire and flames, initial encounter	ICD10CM	SA
**X76.XXXD**	Intentional self-harm by smoke, fire and flames, subsequent encounter	ICD10CM	SA
**X76.XXXS**	Intentional self-harm by smoke, fire and flames, sequela	ICD10CM	SA
**X77.1XXA**	Intentional self-harm by hot tap water, initial encounter	ICD10CM	SA
**X77.2XXA**	Intentional self-harm by other hot fluids, initial encounter	ICD10CM	SA
**X77.3XXA**	Intentional self-harm by hot household appliances, initial encounter	ICD10CM	SA
**X77.8XXA**	Intentional self-harm by other hot objects, initial encounter	ICD10CM	SA
**X77.8XXD**	Intentional self-harm by other hot objects, subsequent encounter	ICD10CM	SA
**X77.9**	Intentional self-harm by unspecified hot objects	ICD10CM	SA

**X77.9XXA**	Intentional self-harm by unspecified hot objects, initial encounter	ICD10CM	SA
**X77.9XXD**	Intentional self-harm by unspecified hot objects, subsequent encounter	ICD10CM	SA
**X78.0XXA**	Intentional self-harm by sharp glass, initial encounter	ICD10CM	SA
**X78.0XXD**	Intentional self-harm by sharp glass, subsequent encounter	ICD10CM	SA
**X78.1XXA**	Intentional self-harm by knife, initial encounter	ICD10CM	SA
**X78.1XXD**	Intentional self-harm by knife, subsequent encounter	ICD10CM	SA
**X78.1XXS**	Intentional self-harm by knife, sequela	ICD10CM	SA
**X78.2XXA**	Intentional self-harm by sword or dagger, initial encounter	ICD10CM	SA
**X78.8XXA**	Intentional self-harm by other sharp object, initial encounter	ICD10CM	SA
**X78.8XXD**	Intentional self-harm by other sharp object, subsequent encounter	ICD10CM	SA
**X78.8XXS**	Intentional self-harm by other sharp object, sequela	ICD10CM	SA
**X78.9XXA**	Intentional self-harm by unspecified sharp object, initial encounter	ICD10CM	SA
**X78.9XXD**	Intentional self-harm by unspecified sharp object, subsequent encounter	ICD10CM	SA
**X78.9XXS**	Intentional self-harm by unspecified sharp object, sequela	ICD10CM	SA
**X79.XXXA**	Intentional self-harm by blunt object, initial encounter	ICD10CM	SA
**X79.XXXD**	Intentional self-harm by blunt object, subsequent encounter	ICD10CM	SA
**X80.XXXA**	Intentional self-harm by jumping from a high place, initial encounter	ICD10CM	SA
**X80.XXXD**	Intentional self-harm by jumping from a high place, subsequent encounter	ICD10CM	SA
**X80.XXXS**	Intentional self-harm by jumping from a high place, sequela	ICD10CM	SA
**X81.0XXA**	Intentional self-harm by jumping or lying in front of motor vehicle, initial encounter	ICD10CM	SA
**X81.0XXD**	Intentional self-harm by jumping or lying in front of motor vehicle, subsequent encounter	ICD10CM	SA
**X81.0XXS**	Intentional self-harm by jumping or lying in front of motor vehicle, sequela	ICD10CM	SA
**X81.1XXA**	Intentional self-harm by jumping or lying in front of (subway) train, initial encounter	ICD10CM	SA
**X81.1XXD**	Intentional self-harm by jumping or lying in front of (subway) train, subsequent encounter	ICD10CM	SA
**X81.1XXS**	Intentional self-harm by jumping or lying in front of (subway) train, sequela	ICD10CM	SA
**X81.8XXA**	Intentional self-harm by jumping or lying in front of other moving object, initial encounter	ICD10CM	SA
**X82.0XXA**	Intentional collision of motor vehicle with other motor vehicle, initial encounter	ICD10CM	SA
**X82.0XXD**	Intentional collision of motor vehicle with other motor vehicle, subsequent encounter	ICD10CM	SA
**X82.2XXA**	Intentional collision of motor vehicle with tree, initial encounter	ICD10CM	SA
**X82.2XXD**	Intentional collision of motor vehicle with tree, subsequent encounter	ICD10CM	SA
**X82.2XXS**	Intentional collision of motor vehicle with tree, sequela	ICD10CM	SA
**X82.8XXA**	Other intentional self-harm by crashing of motor vehicle, initial encounter	ICD10CM	SA
**X82.8XXD**	Other intentional self-harm by crashing of motor vehicle, subsequent encounter	ICD10CM	SA
**X83.1XXA**	Intentional self-harm by electrocution, initial encounter	ICD10CM	SA
**X83.2XXA**	Intentional self-harm by exposure to extremes of cold, initial encounter	ICD10CM	SA
**X83.8XXA**	Intentional self-harm by other specified means, initial encounter	ICD10CM	SA
**X83.8XXD**	Intentional self-harm by other specified means, subsequent encounter	ICD10CM	SA
**X83.8XXS**	Intentional self-harm by other specified means, sequela	ICD10CM	SA
**R45.851**	Suicidal ideations	ICD10CM	SI
**V62.84**	Suicidal ideation	ICD9CM	SI

eTable 2. C-SSRS Logistic Regression Odds Ratios

Outcome	Time (Days)	Feature	Odds Ratio	p-value	Lower 95% CI	Upper 95% CI
SA	7	Has Comment	0.98	0.95	0.56	1.72
SA	7	Q1 (Wish to be dead)	4.21	0.05	0.98	18.0
SA	7	Q2 (Suicidal Thoughts)	0.44	0.28	0.1	1.95
SA	7	Q3 (Thoughts W/Method)	1.07	0.86	0.5	2.3
SA	7	Q4 (Intent W/O Plan)	1.15	0.7	0.57	2.31
SA	7	Q5 (Intent W/Plan)	8.25	0.0	2.75	24.75
SA	7	Q6 (Suicidal Behavior)	7.42	0.0	2.65	20.73
SA	30	Has Comment	1.67	0.0	1.17	2.37
SA	30	Q1 (Wish to be dead)	8.8	0.0	3.96	19.58
SA	30	Q2 (Suicidal Thoughts)	1.3	0.55	0.56	3.03
SA	30	Q3 (Thoughts W/Method)	1.01	0.98	0.63	1.6
SA	30	Q4 (Intent W/O Plan)	1.06	0.79	0.69	1.64
SA	30	Q5 (Intent W/Plan)	2.35	0.0	1.45	3.82
SA	30	Q6 (Suicidal Behavior)	2.46	0.0	1.51	4.03
SA	60	Has Comment	1.78	0.0	1.3	2.44
SA	60	Q1 (Wish to be dead)	8.21	0.0	4.13	16.33
SA	60	Q2 (Suicidal Thoughts)	1.45	0.32	0.7	2.99
SA	60	Q3 (Thoughts W/Method)	0.86	0.47	0.58	1.29
SA	60	Q4 (Intent W/O Plan)	1.1	0.63	0.75	1.61
SA	60	Q5 (Intent W/Plan)	1.98	0.0	1.31	2.99
SA	60	Q6 (Suicidal Behavior)	2.59	0.0	1.69	3.98
SA	90	Has Comment	1.68	0.0	1.27	2.22
SA	90	Q1 (Wish to be dead)	8.06	0.0	4.45	14.58
SA	90	Q2 (Suicidal Thoughts)	1.42	0.27	0.76	2.64
SA	90	Q3 (Thoughts W/Method)	0.79	0.2	0.56	1.13
SA	90	Q4 (Intent W/O Plan)	1.15	0.42	0.82	1.62
SA	90	Q5 (Intent W/Plan)	1.74	0.0	1.21	2.49
SA	90	Q6 (Suicidal Behavior)	3.13	0.0	2.13	4.58
SA	180	Has Comment	1.61	0.0	1.26	2.06
SA	180	Q1 (Wish to be dead)	6.08	0.0	3.58	10.31
SA	180	Q2 (Suicidal Thoughts)	1.81	0.1	1.03	3.15

© 2022 Wilimitis D et al. *JAMA Network Open.*

SA	180	Q3 (Thoughts W/Method)	0.87	0.6	0.64	1.19
SA	180	Q4 (Intent W/O Plan)	1.09	0.9	0.81	1.47
SA	180	Q5 (Intent W/Plan)	1.59	0.04	1.16	2.16
SA	180	Q6 (Suicidal Behavior)	2.75	0.0	2.0	3.8
SI	7	Has Comment	1.17	0.18	0.93	1.46
SI	7	Q1 (Wish to be dead)	14.72	0.0	9.68	22.37
SI	7	Q2 (Suicidal Thoughts)	1.9	0.0	1.22	2.97
SI	7	Q3 (Thoughts W/Method)	0.99	0.95	0.76	1.29
SI	7	Q4 (Intent W/O Plan)	1.23	0.11	0.95	1.59
SI	7	Q5 (Intent W/Plan)	1.38	0.02	1.06	1.8
SI	7	Q6 (Suicidal Behavior)	1.36	0.02	1.06	1.76
SI	30	Has Comment	1.54	0.0	1.31	1.8
SI	30	Q1 (Wish to be dead)	10.59	0.0	7.98	14.04
SI	30	Q2 (Suicidal Thoughts)	2.04	0.0	1.51	2.76
SI	30	Q3 (Thoughts W/Method)	1.07	0.47	0.89	1.3
SI	30	Q4 (Intent W/O Plan)	1.06	0.51	0.88	1.28
SI	30	Q5 (Intent W/Plan)	1.2	0.06	0.99	1.45
SI	30	Q6 (Suicidal Behavior)	1.56	0.0	1.31	1.87
SI	60	Has Comment	1.57	0.0	1.36	1.82
SI	60	Q1 (Wish to be dead)	7.68	0.0	5.93	9.95
SI	60	Q2 (Suicidal Thoughts)	2.37	0.0	1.8	3.13
SI	60	Q3 (Thoughts W/Method)	1.3	0.0	1.09	1.54
SI	60	Q4 (Intent W/O Plan)	0.96	0.59	0.81	1.13
SI	60	Q5 (Intent W/Plan)	1.04	0.63	0.88	1.24
SI	60	Q6 (Suicidal Behavior)	1.82	0.0	1.55	2.15
SI	90	Has Comment	1.77	0.0	1.54	2.03
SI	90	Q1 (Wish to be dead)	6.84	0.0	5.38	8.7
SI	90	Q2 (Suicidal Thoughts)	2.31	0.0	1.79	3.0
SI	90	Q3 (Thoughts W/Method)	1.26	0.01	1.07	1.49
SI	90	Q4 (Intent W/O Plan)	0.99	0.94	0.85	1.17
SI	90	Q5 (Intent W/Plan)	1.03	0.72	0.87	1.22
SI	90	Q6 (Suicidal Behavior)	1.94	0.0	1.66	2.26
SI	180	Has Comment	1.88	0.0	1.65	2.14
SI	180	Q1 (Wish to be dead)	6.57	0.0	5.31	8.15
SI	180	Q2 (Suicidal Thoughts)	2.28	0.0	1.8	2.87

© 2022 Wilimitis D et al. *JAMA Network Open.*

SI	180	Q3 (Thoughts W/Method)	1.21	0.02	1.03	1.41
SI	180	Q4 (Intent W/O Plan)	1.04	0.64	0.89	1.21
SI	180	Q5 (Intent W/Plan)	0.96	0.57	0.82	1.12
SI	180	Q6 (Suicidal Behavior)	2.0	0.0	1.74	2.31

© 2022 Wilimitis D et al. *JAMA Network Open.*

eTable 3. Discrimination Metrics by Risk Threshold Across All Time Periods

Model	Time (Days)	Risk Percentile Cutoff	Specificity (%)	Sensitivity (%)	PPV (%)	NPV (%)	Outcome
C-SSRS TIERS	7	RED	97.6	63.1	1.8	100	SA
C-SSRS TIERS	7	ORANGE	97.1	66.7	1.6	100	SA
C-SSRS TIERS	7	YELLOW	95.5	72.6	1.1	100	SA
C-SSRS TIERS	30	RED	97.7	53.7	3.8	99.9	SA
C-SSRS TIERS	30	ORANGE	97.2	60	3.5	99.9	SA
C-SSRS TIERS	30	YELLOW	95.6	69.3	2.6	99.9	SA
C-SSRS TIERS	60	RED	97.7	50	4.7	99.9	SA
C-SSRS TIERS	60	ORANGE	97.2	55.9	4.4	99.9	SA
C-SSRS TIERS	60	YELLOW	95.6	65.1	3.2	99.9	SA
C-SSRS TIERS	90	RED	97.7	49.7	6.1	99.8	SA
C-SSRS TIERS	90	ORANGE	97.3	54.8	5.6	99.9	SA
C-SSRS TIERS	90	YELLOW	95.6	63.5	4.1	99.9	SA
C-SSRS TIERS	180	RED	97.8	44.2	7.8	99.8	SA
C-SSRS TIERS	180	ORANGE	97.3	49.8	7.4	99.8	SA
C-SSRS TIERS	180	YELLOW	95.7	58	5.5	99.8	SA
C-SSRS REGRESSION	7	50	38.2	86.9	0.1	100	SA
C-SSRS REGRESSION	7	75	57.3	82.1	0.1	100	SA
C-SSRS REGRESSION	7	90	76.2	79.8	0.2	100	SA
C-SSRS REGRESSION	7	95	76.2	79.8	0.2	100	SA
C-SSRS REGRESSION	7	99	98.9	27.4	1.7	99.9	SA
C-SSRS REGRESSION	30	50	37.9	85.4	0.2	99.9	SA
C-SSRS REGRESSION	30	75	56.8	82	0.3	99.9	SA
C-SSRS REGRESSION	30	90	76	76.6	0.5	99.9	SA
C-SSRS REGRESSION	30	95	95.1	73.2	2.5	100	SA
C-SSRS REGRESSION	30	99	99	24.4	4.1	99.9	SA
C-SSRS REGRESSION	60	50	37.9	82.7	0.3	99.9	SA
C-SSRS REGRESSION	60	75	56.9	76.5	0.4	99.9	SA
C-SSRS REGRESSION	60	90	76.1	71.3	0.7	99.9	SA

© 2022 Wilimitis D et al. *JAMA Network Open.*

C-SSRS REGRESSION	60	95	95.1	68	3.1	99.9	SA
C-SSRS REGRESSION	60	99	99.1	25.4	5.7	99.8	SA
C-SSRS REGRESSION	90	50	37.9	82.6	0.4	99.9	SA
C-SSRS REGRESSION	90	75	57.1	75.6	0.5	99.9	SA
C-SSRS REGRESSION	90	90	76.2	68.8	0.9	99.9	SA
C-SSRS REGRESSION	90	95	95.2	66	3.9	99.9	SA
C-SSRS REGRESSION	90	99	99.1	24.4	7.2	99.8	SA
C-SSRS REGRESSION	180	50	37.9	81.9	0.6	99.8	SA
C-SSRS REGRESSION	180	75	56.8	73.9	0.7	99.8	SA
C-SSRS REGRESSION	180	90	75.9	66.7	1.2	99.8	SA
C-SSRS REGRESSION	180	95	95.2	59.7	5.1	99.8	SA
C-SSRS REGRESSION	180	99	99	21.8	8.7	99.7	SA
VSAIL	7	50	49.1	86.9	0.1	100	SA
VSAIL	7	75	75	48.8	0.1	100	SA
VSAIL	7	90	88.7	33.3	0.2	99.9	SA
VSAIL	7	95	95	15.5	0.2	99.9	SA
VSAIL	7	99	99	8.3	0.6	99.9	SA
VSAIL	30	50	49.1	87.3	0.3	100	SA
VSAIL	30	75	75	50.2	0.3	99.9	SA
VSAIL	30	90	88.8	28.8	0.4	99.9	SA
VSAIL	30	95	95	17.1	0.6	99.9	SA
VSAIL	30	99	99	11.7	2	99.8	SA
VSAIL	60	50	49.2	86.4	0.4	99.9	SA
VSAIL	60	75	75.1	51.5	0.5	99.9	SA
VSAIL	60	90	88.8	29.4	0.6	99.8	SA
VSAIL	60	95	95	17.3	0.8	99.8	SA
VSAIL	60	99	99	11	2.5	99.8	SA
VSAIL	90	50	49.2	87.6	0.5	99.9	SA
VSAIL	90	75	75.1	51.7	0.6	99.8	SA
VSAIL	90	90	88.8	29.2	0.8	99.8	SA
VSAIL	90	95	95	16.9	1	99.7	SA

© 2022 Wilimitis D et al. *JAMA Network Open.*

VSAIL	90	99	99	9.6	2.8	99.7	SA
VSAIL	180	50	49.2	88.7	0.7	99.9	SA
VSAIL	180	75	75.1	56.2	1	99.8	SA
VSAIL	180	90	88.8	34.8	1.3	99.7	SA
VSAIL	180	95	95	19.1	1.6	99.6	SA
VSAIL	180	99	99	9.5	4.1	99.6	SA
LASSO	7	50	49.9	89.3	0.1	100	SA
LASSO	7	75	75	83.3	0.2	100	SA
LASSO	7	90	90	79.8	0.6	100	SA
LASSO	7	95	95	76.2	1.1	100	SA
LASSO	7	99	99	33.3	2.3	100	SA
LASSO	30	50	50.1	92.2	0.3	100	SA
LASSO	30	75	75.1	82.9	0.6	100	SA
LASSO	30	90	90.1	79	1.3	100	SA
LASSO	30	95	95.1	75.6	2.6	100	SA
LASSO	30	99	99	21	3.6	99.9	SA
LASSO	60	50	47.1	91.2	0.4	100	SA
LASSO	60	75	75.1	82.4	0.7	99.9	SA
LASSO	60	90	90.1	75	1.7	99.9	SA
LASSO	60	95	95.1	69.9	3.2	99.9	SA
LASSO	60	99	99	19.5	4.4	99.8	SA
LASSO	90	50	48.9	91	0.5	99.9	SA
LASSO	90	75	75.2	81.5	1	99.9	SA
LASSO	90	90	90.2	72.8	2.2	99.9	SA
LASSO	90	95	95.2	66.9	4	99.9	SA
LASSO	90	99	99.1	18.8	5.6	99.8	SA
LASSO	180	50	50.2	93.4	0.8	99.9	SA
LASSO	180	75	75.2	80.9	1.4	99.9	SA
LASSO	180	90	90.3	70.4	3	99.9	SA
LASSO	180	95	95.2	60.9	5.2	99.8	SA
LASSO	180	99	99.1	18.7	8	99.6	SA

© 2022 Wilimitis D et al. *JAMA Network Open.*

AVG	7	50	50	88.1	0.1	100	SA
AVG	7	75	75	84.5	0.2	100	SA
AVG	7	90	90	79.8	0.6	100	SA
AVG	7	95	95.1	77.4	1.1	100	SA
AVG	7	99	99	35.7	2.5	100	SA
AVG	30	50	49.9	90.2	0.3	100	SA
AVG	30	75	75.1	84.4	0.6	100	SA
AVG	30	90	90.1	79.5	1.4	100	SA
AVG	30	95	95.1	73.2	2.5	100	SA
AVG	30	99	99	21	3.6	99.9	SA
AVG	60	50	50	88.6	0.4	99.9	SA
AVG	60	75	75.1	83.1	0.8	99.9	SA
AVG	60	90	90.1	74.6	1.7	99.9	SA
AVG	60	95	95.1	68	3.1	99.9	SA
AVG	60	99	99	22.1	5	99.8	SA
AVG	90	50	50.1	90.2	0.5	99.9	SA
AVG	90	75	75.1	80.6	1	99.9	SA
AVG	90	90	90.2	71.6	2.1	99.9	SA
AVG	90	95	95.2	66	3.9	99.9	SA
AVG	90	99	99.1	22.2	6.6	99.8	SA
AVG	180	50	49.5	91.6	0.8	99.9	SA
AVG	180	75	75.2	81.5	1.4	99.9	SA
AVG	180	90	90.1	69.5	2.9	99.9	SA
AVG	180	95	95.2	59.7	5.1	99.8	SA
AVG	180	99	99.1	21.2	9.1	99.7	SA
WEIGHTED_AVG	7	50	48	96.4	0.1	100	SA
WEIGHTED_AVG	7	75	75	85.7	0.2	100	SA
WEIGHTED_AVG	7	90	90	81	0.6	100	SA
WEIGHTED_AVG	7	95	94.8	75	1	100	SA
WEIGHTED_AVG	7	99	98.9	27.4	1.8	99.9	SA
WEIGHTED_AVG	30	50	48	94.1	0.3	100	SA

© 2022 Wilimitis D et al. *JAMA Network Open.*

WEIGHTED_AVG	30	75	75	82	0.6	100	SA
WEIGHTED_AVG	30	90	90.1	77.6	1.3	100	SA
WEIGHTED_AVG	30	95	95	70.2	2.3	99.9	SA
WEIGHTED_AVG	30	99	99	24.4	4.1	99.9	SA
WEIGHTED_AVG	60	50	48.1	94.5	0.4	100	SA
WEIGHTED_AVG	60	75	75	81.6	0.7	99.9	SA
WEIGHTED_AVG	60	90	90.1	74.3	1.7	99.9	SA
WEIGHTED_AVG	60	95	95	66.5	2.9	99.9	SA
WEIGHTED_AVG	60	99	99.1	25.4	5.7	99.8	SA
WEIGHTED_AVG	90	50	48.1	94.4	0.5	100	SA
WEIGHTED_AVG	90	75	75.1	81.2	1	99.9	SA
WEIGHTED_AVG	90	90	90.2	71.6	2.1	99.9	SA
WEIGHTED_AVG	90	95	95	64	3.7	99.9	SA
WEIGHTED_AVG	90	99	99.1	24.4	7.2	99.8	SA
WEIGHTED_AVG	180	50	48.2	94.9	0.8	100	SA
WEIGHTED_AVG	180	75	75.1	80.5	1.4	99.9	SA
WEIGHTED_AVG	180	90	90.2	66.9	2.9	99.8	SA
WEIGHTED_AVG	180	95	95.1	58.6	4.8	99.8	SA
WEIGHTED_AVG	180	99	99	21.8	8.7	99.7	SA
C-SSRS TIERS	7	RED	97.8	47.4	10	99.7	SI
C-SSRS TIERS	7	ORANGE	97.4	52.9	9.3	99.8	SI
C-SSRS TIERS	7	YELLOW	95.8	65.3	7.4	99.8	SI
C-SSRS TIERS	30	RED	98.1	40.6	20.8	99.2	SI
C-SSRS TIERS	30	ORANGE	97.6	46.2	19.7	99.3	SI
C-SSRS TIERS	30	YELLOW	96.1	58.5	16	99.5	SI
C-SSRS TIERS	60	RED	98.2	37.7	26.5	98.9	SI
C-SSRS TIERS	60	ORANGE	97.8	43.6	25.5	99	SI
C-SSRS TIERS	60	YELLOW	96.3	54.9	20.5	99.2	SI
C-SSRS TIERS	90	RED	98.3	35.7	29.9	98.7	SI
C-SSRS TIERS	90	ORANGE	97.9	41.4	28.9	98.8	SI
C-SSRS TIERS	90	YELLOW	96.4	52.2	23.3	99	SI

© 2022 Wilimitis D et al. *JAMA Network Open.*

C-SSRS TIERS	180	RED	98.4	32.8	35.3	98.2	SI
C-SSRS TIERS	180	ORANGE	98	37.9	34.1	98.3	SI
C-SSRS TIERS	180	YELLOW	96.6	48.8	28	98.6	SI
C-SSRS REGRESSION	7	50	38	86	0.7	99.8	SI
C-SSRS REGRESSION	7	75	57.2	80.1	1	99.8	SI
C-SSRS REGRESSION	7	90	76.2	74.6	1.6	99.8	SI
C-SSRS REGRESSION	7	95	95.2	68.2	6.8	99.8	SI
C-SSRS REGRESSION	7	99	99.1	16.6	8.8	99.6	SI
C-SSRS REGRESSION	30	50	38.2	83.3	1.7	99.5	SI
C-SSRS REGRESSION	30	75	57.4	75.9	2.2	99.5	SI
C-SSRS REGRESSION	30	90	76.5	68.8	3.5	99.5	SI
C-SSRS REGRESSION	30	95	95.7	61.2	15	99.5	SI
C-SSRS REGRESSION	30	99	99.2	17.4	20.9	99	SI
C-SSRS REGRESSION	60	50	38.2	80.8	2.2	99.1	SI
C-SSRS REGRESSION	60	75	57.3	72.3	2.8	99.2	SI
C-SSRS REGRESSION	60	90	76.7	64.2	4.5	99.2	SI
C-SSRS REGRESSION	60	95	95.9	56.5	19.1	99.2	SI
C-SSRS REGRESSION	60	99	99.3	16.6	27.7	98.6	SI
C-SSRS REGRESSION	90	50	38.3	79.7	2.6	98.9	SI
C-SSRS REGRESSION	90	75	57.6	70.8	3.3	99	SI
C-SSRS REGRESSION	90	90	76.8	62.6	5.3	99	SI
C-SSRS REGRESSION	90	95	96.1	53.3	22.1	99	SI
C-SSRS REGRESSION	90	99	99.2	17.1	31.3	98.3	SI
C-SSRS REGRESSION	180	50	38.3	78.5	3.3	98.5	SI
C-SSRS REGRESSION	180	75	57.5	69.1	4.2	98.6	SI
C-SSRS REGRESSION	180	90	76.9	59.6	6.5	98.6	SI
C-SSRS REGRESSION	180	95	96.2	50.5	26.2	98.6	SI
C-SSRS REGRESSION	180	99	99.3	14.4	37.1	97.7	SI
VSAIL	7	50	49.3	90.4	0.9	99.9	SI
VSAIL	7	75	75.2	59.8	1.2	99.7	SI
VSAIL	7	90	88.8	33.4	1.5	99.6	SI

© 2022 Wilimitis D et al. *JAMA Network Open.*

VSAIL	7	95	95.1	20.8	2.1	99.6	SI
VSAIL	7	99	99	7.3	3.7	99.5	SI
VSAIL	30	50	49.6	90.9	2.2	99.8	SI
VSAIL	30	75	75.5	61	3	99.4	SI
VSAIL	30	90	89	35.1	3.9	99.1	SI
VSAIL	30	95	95.2	20.9	5.1	99	SI
VSAIL	30	99	99.1	7.5	9.3	98.8	SI
VSAIL	60	50	49.7	90	3	99.7	SI
VSAIL	60	75	75.6	60.2	4.1	99.1	SI
VSAIL	60	90	89.1	34.8	5.2	98.8	SI
VSAIL	60	95	95.3	20.7	7	98.6	SI
VSAIL	60	99	99.1	7.2	12.1	98.4	SI
VSAIL	90	50	49.9	89.1	3.5	99.5	SI
VSAIL	90	75	75.7	59.1	4.8	98.9	SI
VSAIL	90	90	89.2	33.9	6.1	98.5	SI
VSAIL	90	95	95.3	20.1	8.1	98.3	SI
VSAIL	90	99	99.1	7	14.2	98.1	SI
VSAIL	180	50	50.1	88.3	4.5	99.4	SI
VSAIL	180	75	75.9	59.1	6.1	98.6	SI
VSAIL	180	90	89.4	34.5	8	98.1	SI
VSAIL	180	95	95.4	20	10.4	97.8	SI
VSAIL	180	99	99.2	6.7	17.5	97.5	SI
LASSO	7	50	46.2	94.8	0.9	99.9	SI
LASSO	7	75	75.3	86	1.8	99.9	SI
LASSO	7	90	90.3	75.4	3.9	99.9	SI
LASSO	7	95	95.3	68.4	7	99.8	SI
LASSO	7	99	99.1	18.7	9.6	99.6	SI
LASSO	30	50	45.6	93.5	2.1	99.8	SI
LASSO	30	75	75.7	82	4.1	99.7	SI
LASSO	30	90	90.8	70.1	8.7	99.6	SI
LASSO	30	95	95.7	61.2	15.1	99.5	SI

© 2022 Wilimitis D et al. *JAMA Network Open.*

LASSO	30	99	99.2	17	21	99	SI
LASSO	60	50	50.5	92.6	3.1	99.7	SI
LASSO	60	75	75.9	79.8	5.4	99.5	SI
LASSO	60	90	91	66.5	11.3	99.4	SI
LASSO	60	95	95.9	56.7	19.2	99.2	SI
LASSO	60	99	99.3	16.1	27.2	98.6	SI
LASSO	90	50	50.8	92.7	3.7	99.7	SI
LASSO	90	75	76.1	78.5	6.4	99.4	SI
LASSO	90	90	91.1	64.4	13	99.2	SI
LASSO	90	95	96	54.3	22	99	SI
LASSO	90	99	99.3	14.9	30.1	98.3	SI
LASSO	180	50	51.1	91	4.7	99.5	SI
LASSO	180	75	76.4	76	7.9	99.2	SI
LASSO	180	90	91.4	61.5	16	98.9	SI
LASSO	180	95	96.2	50.7	26.4	98.6	SI
LASSO	180	99	99.4	14.2	37	97.7	SI
AVG	7	50	50.2	93.5	1	99.9	SI
AVG	7	75	75.3	85.5	1.7	99.9	SI
AVG	7	90	90.3	74.9	3.8	99.9	SI
AVG	7	95	95.3	67.9	6.9	99.8	SI
AVG	7	99	99.1	21.5	11	99.6	SI
AVG	30	50	50.5	90.9	2.2	99.8	SI
AVG	30	75	75.7	81.8	4	99.7	SI
AVG	30	90	90.7	69.9	8.6	99.6	SI
AVG	30	95	95.7	60.8	15	99.5	SI
AVG	30	99	99.2	19.7	24.3	99	SI
AVG	60	50	50.7	89.4	3	99.6	SI
AVG	60	75	75.9	78.6	5.3	99.5	SI
AVG	60	90	91	65.7	11.1	99.4	SI
AVG	60	95	95.9	56.6	19.2	99.2	SI
AVG	60	99	99.3	18.1	30.7	98.6	SI

© 2022 Wilimitis D et al. *JAMA Network Open.*

AVG	90	50	50.8	88.7	3.6	99.5	SI
AVG	90	75	76.1	77.6	6.3	99.4	SI
AVG	90	90	91.1	63.7	12.9	99.2	SI
AVG	90	95	96	54	21.9	99	SI
AVG	90	99	99.3	17.2	34.9	98.3	SI
AVG	180	50	50.9	87.7	4.6	99.4	SI
AVG	180	75	76.3	76.5	7.9	99.2	SI
AVG	180	90	91.4	61.4	16	98.9	SI
AVG	180	95	96.2	50.5	26.3	98.6	SI
AVG	180	99	99.4	16	41.6	97.8	SI
WEIGHTED_AVG	7	50	48.2	96.1	0.9	100	SI
WEIGHTED_AVG	7	75	75.2	85.3	1.7	99.9	SI
WEIGHTED_AVG	7	90	90.3	74.1	3.8	99.9	SI
WEIGHTED_AVG	7	95	95.1	66.9	6.6	99.8	SI
WEIGHTED_AVG	7	99	99.1	16.6	8.8	99.6	SI
WEIGHTED_AVG	30	50	48.5	95.2	2.3	99.9	SI
WEIGHTED_AVG	30	75	75.6	81.4	4	99.7	SI
WEIGHTED_AVG	30	90	90.7	67.4	8.3	99.6	SI
WEIGHTED_AVG	30	95	95.5	59.7	14.2	99.5	SI
WEIGHTED_AVG	30	99	99.2	17.4	20.9	99	SI
WEIGHTED_AVG	60	50	48.7	94.2	3.1	99.8	SI
WEIGHTED_AVG	60	75	75.8	79.7	5.4	99.5	SI
WEIGHTED_AVG	60	90	90.9	64.2	10.9	99.3	SI
WEIGHTED_AVG	60	95	95.7	55.9	18.3	99.2	SI
WEIGHTED_AVG	60	99	99.3	16.6	27.7	98.6	SI
WEIGHTED_AVG	90	50	48.8	93.3	3.6	99.7	SI
WEIGHTED_AVG	90	75	76	77.9	6.3	99.4	SI
WEIGHTED_AVG	90	90	91.1	62	12.5	99.1	SI
WEIGHTED_AVG	90	95	95.8	53.3	20.7	99	SI
WEIGHTED_AVG	90	99	99.2	17.1	31.3	98.3	SI
WEIGHTED_AVG	180	50	49	92.3	4.6	99.6	SI

© 2022 Wilimitis D et al. *JAMA Network Open.*

WEIGHTED_AVG	180	75	76.3	75.6	7.8	99.2	SI
WEIGHTED_AVG	180	90	91.3	58.7	15.3	98.8	SI
WEIGHTED_AVG	180	95	96	50	25.1	98.6	SI
WEIGHTED_AVG	180	99	99.3	14.4	37.1	97.7	SI

© 2022 Wilimitis D et al. *JAMA Network Open.*
